# Ataluren—Promising Therapeutic Premature Termination Codon Readthrough Frontrunner

**DOI:** 10.3390/ph14080785

**Published:** 2021-08-09

**Authors:** Sylwia Michorowska

**Affiliations:** Department of Bioanalysis and Drug Analysis, Faculty of Pharmacy, Medical University of Warsaw, 02-097 Warsaw, Poland; ssolobodowska@wum.edu.pl; Tel.: +48-22-5720946

**Keywords:** ataluren, nonsense mutation, readthrough

## Abstract

Around 12% of hereditary disease-causing mutations are in-frame nonsense mutations. The expression of genes containing nonsense mutations potentially leads to the production of truncated proteins with residual or virtually no function. However, the translation of transcripts containing premature stop codons resulting in full-length protein expression can be achieved using readthrough agents. Among them, only ataluren was approved in several countries to treat nonsense mutation Duchenne muscular dystrophy (DMD) patients. This review summarizes ataluren’s journey from its identification, via first in vitro activity experiments, to clinical trials in DMD, cystic fibrosis, and aniridia. Additionally, data on its pharmacokinetics and mechanism of action are presented. The range of diseases with underlying nonsense mutations is described for which ataluren therapy seems to be promising. What is more, experiments in which ataluren did not show its readthrough activity are also included, and reasons for their failures are discussed.

## 1. Introduction

The underlying cause of a large number of human diseases is the presence of nonsense mutations in the corresponding disease genes e.g., many cases of cystic fibrosis (CF), Duchenne muscular dystrophy (DMD), or Usher syndrome [1,2]. Nonsense mutations are also found in frequently mutated genes in human cancers such as *TP53*, *BRCA1*, *PTEN*, *ARID1A*, *RB1,* or *APC* [3]. Still, new human genes bearing nonsense mutations are being identified e.g., *ALDH3B2* gene [4]. These mutations are a type of point mutations resulting from the transformation of sense codons in the coding region, which are decoded by aminoacyl-tRNAs, into nonsense ones (UAA, UAG, or UGA) promoting translation termination. They are also referred to as premature termination codons (PTCs) [5]. Around 12% of hereditary disease-causing mutations are in-frame nonsense mutations [6].

The presence of a stop codon in the ribosomal A site results in translation termination, which in eukaryotes is mediated by eRF1 and eRF3-GTP proteins. eRF1 recognizes the stop codon within the ribosomal A site and the eRF1-eRF3-GTP ternary termination complex triggers significant ribosomal shifts. Subsequently, eRF3-mediated GTP hydrolysis stimulates eRF1 release activity, resulting in the hydrolysis of the ester linkage between the nascent polypeptide chain and the tRNA in the P site [7]. Dissociation and recycling of the termination complex are facilitated by the ABCE1 ATPase and initiation factors eIF3, eIF1, eIF1A, and eIF3j [8].

The expression of genes containing nonsense mutations potentially leads to the production of truncated proteins with residual or virtually no function, as well as gain-of-function or dominant-negative effects. Sometimes, however, the premature stop codons can be bypassed, and ribosomes may continue translation producing C-terminal extended protein. This phenomenon is referred to as stop codon readthrough and on the one hand can constitute a decoding error, but on the other hand, it may represent a therapeutic solution for some pathologies [9]. The readthrough of premature stop codons was first shown in 1985 using aminoglycosides [10]. Since then, many more aminoglycosides as well as non-aminoglycosidic molecules were shown to have similar activity. Examples include, but are not limited to, gentamicin, geneticin (G418), paromomycin, neomycin, lividomycin, ataluren (PTC124, Translarna^TM^), negamycin, RTC13, RTC14, RTC204, RTC219, GJ071, GJ072, NV2907, NV2909, NV2899, NV2913, clitocine, 2,6-diaminopurine, ELX-02, NV2445, and PTC414. However, some of these compounds are toxic. The adverse side effects limit their potential clinical uses. Interestingly, molecules such as amlexanox and escin not only readthrough premature stop codons but additionally inhibit nonsense-mediated decay (NMD) of mRNA [9]. NMD is a process known to protect cells from the deleterious effects of PTCs through the degradation of mutated mRNA. It is regulated by Upf1, Upf2 (NMD2), and Upf3 proteins. It was suggested that in mammalian cells, Upf1 binds to eRFs and activates NMD. Upf1 subsequently interacts with a Upf2:Upf3 heterodimer bound to a downstream exon junction complex [8]. Significantly lower levels of endogenous mRNA were demonstrated in cell lines bearing nonsense mutations in *CLN1*, *CLN2*, and *CLN3* genes [11]. The reduced levels of mRNA containing premature stop codon would decrease the readthrough efficiency. However, it was also shown that some mutant transcripts do not undergo NMD due to their specific sequence context (e.g., levels of mutant *PEX* gene transcripts at least 40% of those in healthy controls [12]).

The readthrough therapy has many advantages comparing to alternative approaches such as gene augmentation, which enables the introduction of an additional functional wild-type gene using a vector [13]. Namely, no random integration of DNA or alternative splicing is possible. The targeted gene remains under normal endogenous control, tissue specificity, timing, and duration of expression [14]. There is no issue of the size of the causative gene or restrictions in the vector capacity [15]. Finally, readthrough therapy is not gene-specific and thus allows for the treatment of diverse genetic conditions. However, the latter feature has drawbacks too, as the readthrough of other than target PTCs can be induced, resulting in the low expression of normally not expressed proteins with potentially negative consequences. 

So far, only ataluren (marketed as Translarna^TM^) developed by PTC Therapeutics was conditionally approved in the European Union in 2014 for the treatment of ambulatory nonsense mutation DMD patients aged 5 years and older [16,17]. In 2018, the indication was extended to include children from 2 to less than 5 years old that are able to walk [18]. Ataluren is also indicated for the treatment of DMD patients aged 2 years or older in Iceland, Israel, Kazakhstan, Lichtenstein, and Norway and patients aged 5 years or older in Brazil, Chile, Ukraine, and the Republic of Korea [19,20]. In Russia, it was granted marketing approval for DMD in December 2020 [21], whereas in the US, ataluren is an investigational drug [22], which means that it has been approved by the US Food and Drug Administration (FDA) for testing in people, and clinical trial results confirming its safety are awaited. So far ataluren’s application for DMD has been rejected by the FDA due to the questionable results’ reliability, as promising ones were obtained via post hoc statistical and exploratory analyses. Therefore, confirmatory clinical trials are required. Moreover, translarna’s application for marketing authorization to treat cystic fibrosis was withdrawn, as the primary endpoint of the phase 3 study was not met.

To date, this is the first extensive review summarizing the journey of ataluren from its identification via in vitro experiments and clinical trials in DMD, CF, and aniridia to approved therapeutic use in DMD. The review also covers ataluren’s pharmacokinetics and mechanism of action including the most recent reports. Results of both successful and unsuccessful readthrough experiments involving models of a wide range of diseases with underlying nonsense mutations are discussed. Potential factors affecting ataluren’s readthrough efficiency are covered. 

## 2. Main Body of Review

### 2.1. Ataluren’s Identification and Structure

**Figure 1:**
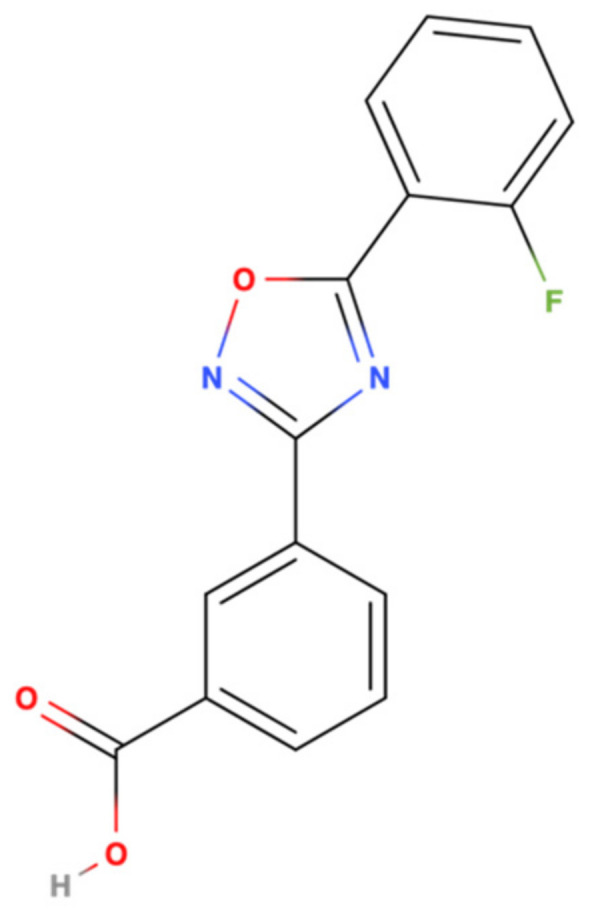
Two-dimensional (2D) structure of ataluren generated using MolView software. The main structural parts are 1,2,4-oxadiazole ring (middle one) connected to fluorobenzene (on the right-hand side) and benzoic acid (on the bottom left-hand side) rings.

Ataluren, known initially as PTC124, is a benzoic acid derivative that was identified in 2007 using two high-throughput screens employing premature TGA luciferase (LUC) reporters in either stably transfected HEK293 cells or rabbit reticulocyte lysates [23]. This low molecular weight compound (284.24 Da), systematically named 3-[5-(2-fluorophenyl)-[1,2,4]oxadiazol-3-yl]-benzoic acid is not optically active and has no structural similarity to aminoglycosides. Its anhydrous, free carboxylic acid form has low solubility in water (<1 μg/mL) [23]. Its structure is shown in Figure 1 below. 

### 2.2. First Readthrough Experiments

The first experiments proving readthrough properties of ataluren were performed using the LUC gene harboring a premature stop codon at threonine 190, replacing ACA with a TAA, TAG, or TGA. The LUC reporter system was used in both cell-based (stably transfected HEK293 cells) and cell-free translation assays (HeLa cytoplasmic extract).

PTC124 promoted dose-dependent readthrough of all three nonsense codons, with the highest efficiency at UGA [23]. The minimum concentration required was in a range of 2.8–28 ng/mL, while a value of approximately 852 ng/mL resulted in the maximum readthrough activity. All these concentrations were lower than those needed in case of gentamicin, indicating a greater potency of PTC124 [23]. 

Subsequent tests on primary muscle cells (myotubes) from humans (DMD patients with premature stop codons TGA and TAG) and *mdx* mice expressing dystrophin nonsense alleles were performed. Immunocytochemical analyses employing an antibody recognizing a carboxyterminal dystrophin epitope revealed readthrough of dystrophin mRNA premature nonsense codons at all concentrations tested (0.5–10 μg/mL). Importantly, the protein produced was located at the myofiber membrane, ensuring muscle structural integrity. The most efficient readthrough was observed at the PTC124 concentration of 5 μg/mL with no further increase at 10 μg/mL [23]. Similarly, Western blot analyses on myoblasts of *mdx* mice also showed the dose-dependent expression of full-length dystrophin in cells treated with PTC124 (0.6–3 μg/mL) [24].

Next, the pharmacological activity of PTC124 in *mdx* mice was assessed using multiple endpoints. For 2–8 weeks, animals were subjected to three different treatment regimens (oral, intraperitoneal injections, and combination of the two) aiming at maintaining target plasma concentration of 5–10 μg/mL. Western blot analyses of *mdx* mouse muscles (quadriceps and tibialis anterior (TA)) showed the full-length dystrophin levels to be approximately 20–25% that of wild-type mice, which may be enough to alleviate the symptoms of DMD patients [23,25]. PTC124 treatment increased the force per cross-sectional area of extensor digitorum longus muscles and resulted in partial protection against contraction-induced injury, which is the best predictor of long-term therapeutic outcome in dystrophic muscles of *mdx* mice and, most likely, in DMD patients. What is more, PTC124 treatment significantly reduced elevated serum creatine kinase (CK) levels (due to lower muscle fragility) and increased muscle γ-sarcoglycan levels. Immunohistological analyses of striated muscles confirmed proper membrane localization in TA, diaphragm, and heart [23]. 

The ability of ataluren to suppress *mdx* nonsense mutations in mice and cultured myotubes was independently confirmed in another study. It was shown that single-dose intramuscular injection of 0.2 mg of PTC124 into TA muscles of *mdx* mice induced the expression of full-length dystrophin, as confirmed by Western blot analyses. Its level was around 5% that of wild-type muscle. Dystrophin was detected along the entire length of the TA muscle, although its distribution throughout the cross-sectional area of the muscle section was rather patchy. Activity of the expressed full-length dystrophin was confirmed by immunoassays of PTC124-treated TA muscles with primary anti-β-dystroglycan antibodies. Clusters of fibers, shown previously to be dystrophin-positive, produced also bright β-dystroglycan staining, confirming the restoration of functionally active dystrophin. Additionally, the therapeutic potential of PTC124 for DMD after systematic administration was shown using young *mdx* mice injected intraperitoneally with 30 mg/kg PTC124 every 5 days over a period of 4 weeks. In all isolated muscles (quadriceps, TA, gastrocnemius, biceps, diaphragm, and heart), dystrophin-positive fibers were detected by immunostaining. Improvement in muscle strength and overall body coordination and strength was shown using the forelimb grip strength test and the four-limb hanging wire test, respectively. Interestingly, the regeneration of these muscles was also demonstrated by the presence of central nuclei in the dystrophin-positive fibers. Furthermore, serum CK levels were significantly decreased. Altogether, these analyses indicated that a low amount of full-length dystrophin improves the overall muscle pathology characteristic of *mdx* mice [24].

Translational readthrough of the premature stop codons by PTC124 was also demonstrated in a mouse line carrying a knockout endogenous *Cftr* locus and expressing human *CFTR* cDNA containing the G542X premature nonsense mutation (Cftr-/-*hCFTR*-G542X mouse line). While the primary cause of death in CF patients is lung infections, intestinal blockages are observed in CF mouse models instead due to the lack of a functional protein, cystic fibrosis transmembrane conductance regulator (CFTR). Murine CFTR is normally found at the epithelial surface of intestinal glands in the colon, ileum, and jejunum. It was shown that once-daily subcutaneous injections of 15, 30, and 60 mg/kg or oral administration of 0.3 or 0.9 mg/mL of PTC124 in a liquid diet for 14–21 days suppressed the G542X nonsense mutation, restoring significant levels of functional human CFTR protein. Immunochemical staining of mouse intestinal tissues confirmed the dose-dependent expression of CFTR protein at the apical surface of intestinal glands in the duodenum. The functional activity of the protein was demonstrated by detecting increased cAMP-stimulated transepithelial chloride currents (up to 29% and 24% of the wild-type after once-daily subcutaneous injections of 60 mg/kg PTC124 or a liquid diet containing 0.9 mg/mL PTC124, respectively) in intestinal tissue. The highest subcutaneous dose resulted in PTC124 peak serum concentration of 10 μg/mL 15 min after injection, which is the higher value of the optimal plasma concentration range as determined in studies with *mdx* mice. Interestingly, the data suggested a low level of endogenous readthrough of the premature stop codon [26]. 

### 2.3. Mechanism of Action

The initial experiments involving the stable cell line LUC reporter assay showed that readthrough occurred at each of the nonsense codons, with maximum activity at UGA and minimum at UAA [23,27]. The efficiency was affected by the premature stop codon context, yielding the highest results with pyrimidine (especially cytosine, C) in the +1 position. Interestingly, the UGA-G termination context was also characterized by high readthrough levels [23]. The induction of specific premature stop codon readthrough was also shown ex vivo in the phase 1 studies using a cell-based luciferase reporter system. Pooled plasma samples of PTC124-treated healthy subjects were added to the culture medium of human embryonic kidney (HEK293) cells that were stably transfected with a firefly luciferase reporter gene bearing a TGA premature stop codon at position 190. Chemiluminescence signals confirmed specific premature stop codon readthrough [28].

PTC124 was shown not to alter mRNA levels, indicating no effects on neither transcription nor mRNA stability. Lack of interferences with NMD suggests that termination efficiency is modulated at premature nonsense codons and confirms the translation-specific action [23]. The RT-PCR analyses of intestinal tissues of *Cftr*-/- *hCFTR*-G542X mice treated with 0.9 mg/mL PTC124 in the liquid diet showed a comparable level of mRNA as compared to the untreated mice, confirming the selective promotion of translation readthrough by PTC124 [26]. In a phase 2 study involving adult CF patients (NCT00237380), no change in the mean proportion of *CFTR* mRNA isolated from the nasal epithelium of treated patients relative to wild-type *CFTR* mRNA was observed during the treatment with 4, 4, 8 and 10, 10, 20 mg/kg PTC124 (as measured by real-time PCR). This confirms that ataluren does not increase gene transcription or mRNA stability [29]. However, in other rare cases (HeLa cells transfected with construct bearing nonsense mutation of *MUT* gene or zebrafish model bearing nonsense mutation of *CHM* gene), increases in mRNA level were observed after ataluren treatment [30,31]. 

Additionally, neither frameshifts mutations nor multiple sequential premature stop codons were affected by ataluren [23]. 

Importantly, two-dimensional gel electrophoresis on luciferase-transfected HEK293 cells and Western blot analyses on multiple tissues isolated from rats, dogs, and humans (peripheral blood mononuclear cells, PBMCs) demonstrated ataluren’s selectivity toward premature nonsense codons over normal termination codons, even at drug exposure levels higher that those achieving maximal activity (200 mg/kg) [23]. Western blot analyses of high-abundance rats’ (vimentin, a-actin, U1 snRNP A, and cofilin) and dogs’ (GAPDH, B-actin, cofilin) proteins evaluating each stop codon type (TGA, TAA, and TAG) showed no elongated transcripts in tissues of animals given PTC124 orally at 1500 mg/kg for 14 days [32]. No nonspecific stop codon readthrough was either observed in phase 1 studies, in which pooled PBMC and plasma samples of healthy volunteers were subjected to Western blot analyses. Neither untreated nor PTC124-treated subjects showed elongated forms of the three proteins: C reactive protein, β2 microglobulin, and cystatin C, each bearing a different normal stop codon (TGA, TAA, and TAG, respectively) and a second downstream in-frame stop codon in the 3′-untranslated region [28]. 

Assays with six strains of Gram + and − bacteria showed no antimicrobial activity of ataluren up to the concentration of 880 μM [27]. 

RNA footprinting experiments with HeLa cell ribosomes treated with PTC124 and dimethyl sulfate or kethoxal before primer extension revealed changes at the conserved sites of the large rRNA subunit modulating nonsense suppression activity in prokaryotic systems. This indicates a distinct mechanism of action, since aminoglycosides interact with the small rRNA subunit [27].

The effect of the simultaneous use of aminoglycosides with ataluren was assessed using in vitro luciferase assay post hoc of phase 3 study (NCT00803205) on CF patients. Tobramycin and gentamicin (having a bacterial ribosomal binding mechanism of action) co-incubated with ataluren reduced the ataluren-induced readthrough of premature stop codons. The suspected interference of these two antibiotics with ataluren’s mechanism of action was supported by the disparity observed in multiple endpoints between the subgroup of patients not taking inhaled tobramycin and the subgroup of patients taking it chronically [33]. 

In another experiment performed post hoc in the same study, it was confirmed that ataluren does not affect the antibacterial activity of tobramycin [33].

The induction of readthrough is usually measured using animals, intact cells, or crude cell extracts. As a result, a compound being investigated can promote readthrough directly via binding to one or more components of the protein synthesis machinery or even indirectly either by inhibiting NMD mRNA decay or by impacting the processes affecting the cellular activity levels of protein synthesis machinery components. 

A new, highly purified in vitro assay (PURE-LITE) was developed to measure exclusively direct nonsense suppressor-induced readthrough. It is composed of purified eukaryotic 80S ribosomes programmed with variants of the cricket paralysis virus internal ribosome entry site mRNA, aminoacylated tRNA isoacceptors, elongation factors eEF1A and eEF2, as well as release factors eRF1 and eRF3. Thus, the synthesis of complete proteins occurs in a cell-free system completely lacking initiation factors [34]. It was shown that both aminoglycosides and ataluren-like compounds induce readthrough by a direct effect on the protein synthesis machinery. However, aminoglycosides exert this effect by binding to the ribosome at a single tight site, while ataluren-like compounds do that at multiple sites. In this case, the binding is weaker and induces a slower change in the protein synthesis apparatus that permits readthrough [35]. The saturation dose-dependent behavior observed in this study contrasts with the bell-shaped one observed in live cells (discussed later) [8]. This decrease in readthrough activity at higher concentrations may be caused by interactions of ataluren with cellular components not present in the in vitro assay used in the PURE-LITE study [35]. In the subsequent experiments, using rate measurements of elementary steps in a single eukaryotic translation cycle, it was shown that ataluren and the aminoglycoside G418 stimulate premature stop codons readthrough via orthogonal mechanisms. G418 was found to mainly increase functional near-cognate tRNA mispairing resulting from tight binding to its primary site on the decoding center of the eukaryotic ribosome with the subsequent continuation of synthesis, while ataluren was shown to exclusively inhibit release factor-dependent termination of protein synthesis via more than one site interaction. PTC124 was suggested to inhibit eRF1/eRF3-dependent peptidyl-tRNA hydrolysis activity, as no ataluren readthrough activity in the absence of eRF1/eRF3 was observed or no effects on the measured rate constants for near-cognate ternary complex binding, peptide bond formation, and translocation were demonstrated. Intriguingly, the ataluren EC_50_ values in these assays were around 10 to 30 times higher than ataluren concentrations typically used in growth media in experiments with cells and tissue cultures. However, it should be noted that ataluren being highly hydrophobic is easily taken up by cells, and the cellular concentration is for sure much higher than that of cell culture medium. Taking into account the low toxicity of ataluren, it was suggested that the development of new readthrough compounds with exclusive effect on termination should be a priority [36].

### 2.4. Pharmacokinetics

#### 2.4.1. Absorption

A phase 1 study enrolling seven healthy males (aged 19 to 50) showed greater than 55% bioavailability [8]. Another phase 1 study indicated dose proportionality for C max and AUC and no significant sex effects on any pharmacokinetic parameter. In a single increasing-dose study, it was shown that the peak concentration (t_max_) occurred after 1–3 h of administration and the mean half-life (t_1/2_) was in the range of 3 to 6 h, while in a multiple-dose study, these parameters were in the range of 2 to 4 and 2 to 6 h, respectively. Plasma concentrations exceeding those with preclinically confirmed activity (2–10 μg/mL) were safely achieved [28]. No significant difference between the fasted and fed state was shown with just a modest delay in drug absorption after a high-fat, high-calorie meal. Though, it was recommended to take the drug within 30 min of a meal, as the plasma concentrations were better sustained when the drug was taken with food [28]. Diurnal variations were observed with greater mean C_max_ (up to 43%) and mean AUC_0–12_ (up to 50%) values following the evening dose as compared to the morning dose. They could be explained by circadian or sleep-related changes in gastrointestinal motility, cardiac output, hormone levels, renal, hepatic, or mesenteric blood flow, and tissue fluid shifts [28]. In a phase 2 study (NCT00803205) on CF patients taking PTC124 at two dosage regimens (4, 4, 8 and 10, 10, 20 mg/kg) for 14 days, rapid oral absorption was shown with the dose-proportional increase in pharmacokinetic parameters, which were not affected by sex [29]. Similar results were obtained in chronic phase 2 study on adults (NCT00351078) as well as in phase 2 study on CF children (NCT00458341), indicating no need for age-adjusted dosing for CF children [37,38]. 

#### 2.4.2. Sigmoidal Dose–Response Curve

The production of dystrophin in cultured myotubes isolated from *mdx* mice exposed to ataluren showed a bell-shaped concentration–response curve [23]. A similar response was observed in experiments with cultured myotubes isolated from DMD patients [39] as well as the zebrafish DMD model [40]. These observations were further confirmed in a phase 2b study (NCT00592553) in DMD patients, where 20, 20, 40 mg/kg ataluren dosage regimen resulted in negligible differences over placebo, while a lower dose regimen (10, 10, 20 mg/kg) produced much better responses. What is more, further analysis of the ataluren concentration revealed that patients receiving higher doses but having plasma concentrations in the range observed with the lower dosage regimen experienced better outcomes (lower mean decline in 6-Minute Walk Distance (6MWD) and better performance of timed function tests (TFTs)) than patients with higher concentrations [41]. 

In a phase 2 study (CF patients; NCT00237380), the assessed parameters did not show the dose-dependent response possibly because both dosage regimens (4, 4, 8 mg/kg and 10, 10, 20 mg/kg) resulted in the upper end of a sigmoidal dose–response curve [29]. In a phase 2 study on CF children (NCT00458341), there was a somewhat greater degree of hyperpolarization at a higher dose level (10, 10, and 20 mg/kg), though the other endpoint (the total chloride transport) did not show a dose-dependent response, suggesting again that both dose levels may be at the upper end of a sigmoidal dose–response curve [38]. On the other hand, in a chronic phase 2 study on CF adults (NCT00351078), numerically but not a statistically greater total chloride transport was observed at the lower dose level (4, 4, 8 mg/kg) than at the higher one (10, 10, 20 mg/kg). This observation could be explained by the patients’ allocation pattern used, in which patients with the best response to a lower dosage regimen in the previous short-term study were assigned to a lower dose in the current study, while those previously responding better to higher doses were assigned to a higher dosage regimen. As a consequence, the most responsive patients may have been assigned to the lowest doses, biasing the effect of dose level on the outcomes [37].

#### 2.4.3. Distribution, Metabolism, and Elimination

In culture cells experiments with fetal bovine serum (5–50% concentration range), it was shown that drug–protein binding does not limit the activity [23,27]. PTC124 pharmacokinetics were described by a one-compartment model [28]. Biodistribution analyses involving heart, diaphragm, TA, quadriceps, gastrocnemius, and biceps brachii muscles of *mdx* mice after a single intraperitoneal injection of 30 mg/kg PTC124 showed rapid uptake of a drug by these tissues with the highest concentration in the heart, followed by gastrocnemius, quadriceps, diaphragm, TA, and biceps brachii muscles [24].

There was neither any drug accumulation nor substantial autoinduction of drug metabolism observed in phase 1 healthy volunteers and phase 2 study CF patients [28,29]. 

Ataluren is primarily metabolized via direct glucuronidation mainly by UGT1A9 and to a lesser extent by UGT1A7, as demonstrated in in vitro experiments with human liver microsomes and recombinant enzymes. The contribution of CYP enzymes to ataluren metabolism was shown to be minimal. Glucuronidation was also demonstrated with human intestinal and kidney microsomes but not with human pulmonary microsomes. Human kidney microsomes were more active than liver microsomes and human intestinal microsomes were the least active, but they were still able to contribute to first-pass metabolism following an oral dose. Ataluren did not inhibit CYP 1A2, 2B6, 2C19, 2D6, and 3A4/5 enzymes activity, but it turned out to be a non-competitive inhibitor of CYP2C8 and a competitive inhibitor of CYP2C9. However, the concentrations of the free ataluren in human plasma after administration of the most typical dosage regimen (10, 10, 20 mg/kg) is much lower than inhibition constants toward these enzymes due to the high protein binding of the drug. What is more, clinically irrelevant induction of CYP2B6 and CYP2C9 activity in cultured primary human hepatocytes was also observed [42].

The single oral administration of [^14^C]-ataluren resulted in approximately 15.2%, 10.0%, 5.2%, 4.3%, and 7.0% of the area under the plasma concentration curve of total radioactivity in mice, male and female rats, dogs, and humans, respectively. Ataluren underwent extensive metabolism after oral administration in mice, rats, dogs, and humans, with phase I metabolism being negligible and glucuronidation being the dominant pathway [43]. Initially, only 1β-isomer of ataluren’s acyl glucuronide is formed. It is known that 1β-isomers of carboxylic acid-containing drugs can rearrange to the 2-, 3-, and 4-isomers, which may induce toxic effects due to their possibility of covalently binding to proteins [44]. However, in vivo (plasma samples of mice, rats, dogs, and one human patient) tests on ataluren, being a benzoic acid derivative, showed that the only detectable and stable ataluren acyl glucuronide metabolite is ataluren-O-1β-acyl glucuronide, confirming no safety concern related to ataluren treatment [45]. 

A study on the pharmacokinetics of ataluren in 24 Japanese and 24 Caucasian healthy male subjects was conducted to evaluate potential ethnicity-related differences. In this phase 1, open-label, single-dose parallel-group study, subjects were administered one out of three dose levels: 5, 10, or 20 mg/kg once daily. Similar, dose-dependent C_max_ values at each dose level were observed for the two study groups. Dose-independent mean half-life values of approximately 3.4 h were also similar in both groups. However, the mean AUC_(0–last)_, which increased with increasing dose, was higher in Caucasians at each of the three dosage regimens. The observed difference was likely due to greater variability of recorded values in Caucasian subjects and a small study population. Overall, the pharmacokinetic profiles in both groups were similar, suggesting no effect of race on the absorption, metabolism, or disposition of ataluren [46].

It was shown that PTC124 is excreted by both hepatic and renal routes [8]. Excretion with urine as the parent drug was low (<2% of the total PTC124 dose) [28]. Single oral administration of [^14^C] ataluren to mice, rats, dogs, and humans resulted in approximately 39%, 17–21%, 12%, and 55% recovery of radioactivity in the urine and 54%, 70–72%, 80%, and 47% in the feces, respectively. The major route of the elimination of ataluren in rats seems to be biliary secretion as evidenced by approximately 10%, 7%, and 82% of the dose recoveries in the urine, feces, and bile, respectively [43]. 

Based on the pharmacokinetics data collected in phase 1 studies, an optimum 3 times a day dosing regimen was suggested. Two lower doses at 6 h intervals during the day and one greater at a 12 h interval overnight, all taken with meals, should sustain target plasms concentrations [28]. 

#### 2.4.4. Palatability

Phase 1 studies showed that administered orally PTC124 water suspension was palatable and well tolerated up to 100 mg/kg/dose [28].

#### 2.4.5. Toxicity

Preclinical safety pharmacology and toxicology studies on rats and dogs showed no obvious toxicity of ataluren [47], which is consistent with its mechanism of action: no effect on a genomic surveillance mechanism—NMD and specificity for premature stop codons [23]. At doses up to 1500 mg/kg given orally to rats and dogs for 28 days, there were no adverse neurological, pulmonary, or cardiovascular effects reported [47]. However, dogs showed signs of adrenalitis [37]. Standard in vitro and in vivo assays showed no mutagenic or genotoxic activity. There was no inhibition of human ether-a-go-go-related gene channel inhibition, indicating no proarrhythmic potential. Moreover, PTC124 was stable in an in vitro model of hepatic clearance employing liver microsomes [47]. The symptomatic adverse effects that were observed in two phase 1 studies were rather mild, including mild headache, dizziness, and gastrointestinal events mainly at doses 150 and 200 mg/kg. Asymptomatic elevations of serum alanine aminotransferase (ALT) and aspartate aminotransferase (AST) levels at doses 200 mg/kg/single dose and 20–50 mg/kg/two times a day went back to normal shortly after the discontinuation of PTC124 administration. Other hepatic parameters such as serum alkaline phosphatase, gamma-glutamyl transferase, lactate dehydrogenase, and bilirubin levels were not affected. There was no evidence of cholestatic nor functional impairment [28]. Analyses of serum creatinine, blood urea nitrogen, and urine showed no signs of nephrotoxicity [28]. Importantly, no significant changes in hematological parameters or ECGs were observed. Based on the collected data, a 100 mg/kg/single dose was found to be the maximum tolerated single dose [28]. 

### 2.5. Phase 1 Studies

The first phase 1 study on seven healthy male volunteers (aged 19 to 50) investigated the absorption, metabolism, and excretion of PTC124 [8].

The other two phase 1 studies (single- and multiple-dose) on 62 healthy volunteers aged 18–30 showed that PTC124 was well tolerated at plasma levels higher than those required for the premature translational termination suppression in cell cultures and animal models [28]. Appropriate volumes of a 150 mg/mL ataluren water suspension were administered orally to yield 3, 10, 30, 100, and 200 mg/kg doses in an initial, single-dose study. Next, volumes of ataluren suspension corresponding to 10, 20, 30, and 50 mg/kg were taken twice per day at mealtimes in the subsequent multiple-dose study [28]. They were co-administered with milk regarding the potential future administration of ataluren to children. The dose range was chosen to achieve and maintain the target plasma concentrations observed in preclinical studies. 

Data collected in phase 1 studies supported the initiation of phase 2 studies involving patients with nonsense-mutation-mediated CT and DMD [28]. However, a noteworthy limitation of these phase 1 studies was the prevalence of Hispanic and African American subjects, while it is known that the majority of CF and DMD patients have Caucasian ancestry [28].

### 2.6. Clinical Studies in Duchenne Muscular Dystrophy

#### 2.6.1. Duchenne Muscular Dystrophy

DMD is a severe, progressive, rare, neuromuscular, X-linked genetic disease affecting up to 0.017% of males. Mutations (mainly deletions, but also point mutations, small insertions, duplications) in the dystrophin gene result in the production of the cytoskeletal protein with little or no function [48,49]. One-third (34%) of all mutations are nonsense mutations [49]. Dystrophin is a 427 kDa plasma membrane protein of muscle fibers that links the intracellular actin skeleton to the extracellular matrix via the dystrophin–glycoprotein complex, thus protecting muscles from stress [8,49]. The proper localization of other components (e.g., sarcoglycans, dystroglycan) of the complex is affected by the functional dystrophin presence [24]. Loss of dystrophin functions results in wasting of dystrophin-negative muscle fibers with subsequent muscle degeneration and functionality loss due to failed regeneration [50,51]. The symptoms include proximal muscle weakness due to progressive muscle degeneration as well as respiratory orthopedic and cardiac complications. The mean age at death is around 19 years [48]. 

One of the models most commonly used in DMD studies is the *mdx* mouse model characterized by the premature termination stop codon (TAA in place of GAA) in the dystrophin gene at 27% of the full-length protein [50,52]. These mice are frequently used in preclinical investigations of novel therapeutics, as they show similar muscle pathology to DMD patients [24,51]. The dystrophin-deficient muscle fibers of *mdx* mice are more susceptible to contraction-induced sarcolemmal rapture [53]. That is why the protection against contraction-induced injury may be a good predictor of long-term therapeutic outcome. Additionally, the molecular marker serum CK can be used to monitor the progression of dystrophin-negative muscle fragility, as its levels positively correlate with progressing muscle damage [51].

#### 2.6.2. Phase 2 and 3 Trials as Well as Observational Studies

From December 2005 to May 2007, a phase 2a open-label, sequential dose-ranging trial (NCT00264888) was conducted in 38 boys (≥5 years of age) with nonsense mutation DMD. Patients were administered ataluren for 28 days, three times a day according to one of the three dosage regimens (4, 4, and 8 mg/kg; 10, 10, and 20 mg/kg or 20, 20, and 40 mg/kg, respectively). The most common nonsense mutation was TGA (58%), whereas TAG and TAA were present in only 21% of patients each. 

The primary endpoint of the study was the expression of full-length dystrophin in the extensor brevis muscle samples obtained via biopsy. More than half (61%) of patients showed increases in dystrophin level due to ataluren treatment in a quantitative immunofluorescence assessment based on dystrophin/spectrin ratio, with the mean change from pre-treatment to post-treatment of 11.0% in dystrophin expression (increase in light intensity relative to baseline). Positive results were additionally confirmed by the qualitative immunofluorescence analysis showing an increase in staining for dystrophin in 33%, 40%, and 25% of patients being administered low, medium, and high doses, respectively. Expressed dystrophin was properly localized at the sarcolemma of the muscle fibers. Immunofluorescence was also used to analyze dystrophin levels in myotubes cell cultures obtained from pre-treatment primary muscle cells of 92% of patients. Exposure to 10 μg/mL ataluren for nine days resulted in an increase in dystrophin expression in all samples, meaning that even non-responders by muscle biopsy had the potential to respond at a cellular level. 

Serum CK levels decreased significantly in 84% of patients treated with medium and high dosage regimes, though no definite dose–response relationship was identified. The decreased levels returned to baseline upon cessation of the treatment, which may additionally prove the pharmacological activity of ataluren.

When it comes to clinical outcomes, no statistically significant changes were observed in upper and/or lower extremity myometry scores (hand grip, elbow flexion, hip abduction, and knee extension) or TFTs (standing from supine, running 10 m, climbing four standard stairs), which is not surprising, taking into account the short time of the treatment period. However, informal improvement in activity and endurance, as well as lower fatigue were observed by parents and teachers during the patients’ treatment.

No relationship between the expression level and any of the following factors—nonsense mutation type, exon location, or corticosteroid use—was identified. 

Two higher dosage regimens resulted in the ataluren plasma concentrations that were in or above the range which was proven to be active in the *mdx* muse model (2–10 μg/mL). 

Laboratory tests, vital signs, electrocardiograms, and physical examinations did not show any signs of serious adverse events or side effects. The most common were gastrointestinal-related events such as flatulence, diarrhea, vomiting, abdominal discomfort or pain, and nausea (58% of patients) [39].

The authors of this study emphasized limitations of currently available methods used to assess changes in dystrophin expression. The usefulness of muscle biopsy dystrophin expression as a biomarker in therapeutic trials targeting small increases in dystrophin level is questionable as samples provide local information of muscle quality, which may not reflect the state of all muscles. What is more, typically before treatment, a sample is taken from one side of the body and from the other one after treatment, which introduces variability. Furthermore, a DMD muscle is often heterogeneous with respect to fibrofatty replacement of muscle and as a consequence, possible sampling errors may occur during a biopsy. Available assays were not very sensitive, robust, or reproducible at quantifying low levels of dystrophin. Different techniques (e.g., immunostaining and Western blot) applied to the same specimen give inconsistent results. Furthermore, using CK as a pharmacodynamic endpoint in DMD is also problematic, as changes in CK are highly variable due to physical activity (exercise-related stress of the body increases the CK levels) [39].

A subsequent phase 2b (February 2008–December 2009) randomized, double-blind, placebo-controlled, multiple-dose, international long-term efficacy, and safe study (NCT00592553) enrolled 174 males (≥5 years). They received ataluren three times a day according to one of the dosage regimens, either 10, 10, and 20 mg/kg or 20, 20, and 40 mg/kg, for 48 weeks. All three premature stop codon types were represented with TGA being the most common (47.7%) followed by TAG (27.6%) and TAA (24.7%). The primary endpoint, which was the change in 6MWD by at least 30 m versus placebo at week 48, was met by the lower dosage regimen group, though it was not statistically significant. Greater and, more importantly, statistically significant differences in the 6MWD were observed between the prespecified baseline 6MWD < 350 m subgroup over the placebo group and the ambulatory decline-phase subgroup (patients aged > 7, on steroids and baseline of 6MWD from 150 m to 80% predicted) over the placebo group comparing to results of the overall population versus the placebo group. The difference in the mean change in 6MWD between the placebo group and the higher dosage regimen group was negligible. The range of standard deviation was high due to variability of the 6MWD over 48 weeks.

Furthermore, it was shown that patients treated with a lower dosage regimen were less likely to lose their walking ability, which is defined as a 10% reduction in 6MDW from baseline, which indicated substantially slower disease progression. 

Clinically meaningful though not statistically significant differences between the placebo group and patients administered a lower dosage regimen were also observed in one of the secondary endpoints, that is TFTs including 4-stair ascend, 4-stair descend, and 10-m walk/run (the differences between ataluren versus the placebo group for mean changes in the stand from supine test were small for both dose levels). Again, greater differences in the TFTs were observed between the ambulatory decline-phase subgroup over the placebo group and even greater for the prespecified baseline 6MWD < 350 m subgroup over the placebo group.

Positive trends favoring a lower dosage regimen of ataluren versus placebo were observed for other secondary endpoints: at-home activity, myometric evaluation of muscle strength (knee flexion and extension, elbow flexion and extension, and shoulder abduction), patient/caregiver-reported accidental fall frequency as well as patient-reported physical functioning. The last outcome was even more pronounced in case of the ambulatory decline-phase subgroup. 

The expression of dystrophin in biceps brachii obtained via biopsy at baseline and from the contralateral around week 36 showed a slightly higher dystrophin/spectrin ratio in the ataluren-treated group versus the placebo group, again with more pronounced results in case of the lower dosage regimen group. However, the results were difficult to interpret due to general sample quality and limitations of the immunofluorescence quantification method. No significant changes in the laboratory or physical parameters were observed. Most ataluren-related adverse events were mild or moderate [41]. 

A subsequent open-label extension phase 3 study (NCT01247207) aimed at assessing the safety and tolerability of ataluren in 160 DMD patients with prior exposure to ataluren was started in November 2010 in Canada and the United States and is still running (the estimated completion date is December 2021). Patients are being given ataluren three times a day (10, 10, 20 mg/kg).

A multicenter, randomized, double-blind, placebo-controlled, phase 3 trial (NCT01826487) was conducted between March 2013 and August 2014 at 54 sites in 18 countries of North America, Europe, the Asia-Pacific region, and Latin America. A total of 230 boys aged 7–16 years with nonsense-mutation DMD received ataluren orally three times daily (10, 10, 20 mg/kg) for 48 weeks. The primary endpoint was the change in 6MWD from baseline to week 48, which is an indicator of disease progression. The least-squares mean change in this parameter was lower for ataluren-treated patients than for the placebo group (−47.7 m and −60.7 m, respectively), resulting in a statistically insignificant 13.0 m difference (lower than 30 m, which is the value prespecified for statistical power). This primary endpoint was also analyzed in prespecified subgroups based on baseline 6MDW, which is reflective of anticipated rates of disease progression over 1 year. The three subgroups were: a subgroup with a 6MWD of less than 300 m (further referred to as lowest 6MWD group), a subgroup with a 6MWD of 300 m or more to less than 400 m (further referred to as medium 6MWD group), and a subgroup with a 6MWD of 400 m or more (further referred to as highest 6MWD group). Stratification by baseline 6MWD is justified, as the sensitivity of this parameter in patients with higher baseline function is decreased, and interpatient variability in patients with a baseline 6MWD of less than 300 m is increased. The statistically significant least-squares mean change for ataluren versus the placebo group was observed in the prespecified medium 6MWD group (−27.0 m vs. −69.9 m), resulting in a 42.9 m difference. Statistically insignificant differences in least-squares mean changes between treated groups and the placebo group were observed for the other two prespecified groups. In case of the lowest 6MWD group, a huge decrease in least-squares mean changes for the treatment group and placebo group was observed, with a value higher by 1 for the placebo group at week 48. The least-squares mean changes in the highest 6MWD group were small, with rather small differences between the treatment group and placebo group, which at week 48 was −9 m. 

The secondary efficacy endpoint was the effect of ataluren on proximal muscle function as assessed by TFTs as well as time to 10% persistent worsening in 6MWD. The ataluren-receiving group was characterized by a lesser decline in physical function compared with the placebo group as measured by these tests. However, the only statistically significant difference was obtained in the four-stair descend test. This effect was more pronounced in the medium 6MWD group of patients.

The North Star Ambulatory Assessment (NSAA), indicating improved functions important in everyday life, numerically but not significantly favored the ataluren group. However, the difference in this treatment effect was statistically significant in the medium 6MWD group. 

Additionally, the prespecified meta-analysis based on data obtained from this trial as well as the data of a subgroup of patients of the phase 2b trial (NCT00592553) meeting the entry criteria of this phase 3 study was performed. Combined 6MWD data showed treatment benefit for ataluren versus the placebo group, while TFTs showed a lesser decline. 

Additional post hoc analyses demonstrated reduced loss of ambulation in patients treated with ataluren comparing to placebo in both phase 2b and phase 3 trials. None of the patients in the medium 6MWD group receiving ataluren lost ambulation after 48 weeks. 

Significantly less deterioration was shown by treated patients than the placebo group in the post hoc composite analysis of TFTs, again with more noticeable results in case of the medium 6MWD group patients. However, the change in time to lose ability to climb and descend four stairs, numerically favoring ataluren, was not statistically significant.

The post hoc analysis of the 17 individual functions of the NSAA demonstrated reduced loss of functions for patients given ataluren compared with those given placebo. A statistically significant 31% reduction of risk of loss of function for the ataluren-treated group was noted, which was again more pronounced in case of the medium 6MWD group. 

The other exploratory efficacy endpoints such as parent-reported health-related quality of life (by the Pediatric Outcomes Data Collection Instrument), the activities of daily living, and disease status survey were not statistically different. 

The drug was well tolerated with most adverse effects being mild to moderate. Only 3% of patients reported serious adverse events, which were considered unrelated to treatment in all cases except one.

One of the important limitations of this study was a relative high percentage of patients with 6MWD baseline equal to or greater than 400 m, which was characterized by a higher range of ambulatory ability, stable state over 48 weeks, and reduced sensitivity of the 6MWD test. Their inclusion may explain the attenuated treatment effect of ataluren observed in this study. The medium 6MWD group, experiencing a transition to ambulatory deterioration, is believed to represent a stage of the disease with the most likely response to dystrophin restoration therapy.

Altogether, the results of this phase 3 trial showed significant treatment benefit of ataluren versus placebo, especially in patients with a baseline 6MWD of 300 m or more to less than 400 m [54].

Meta-analyses of data collected in the two completed randomized controlled trials described above (NCT00592553 and NCT01826487) were used to assess the efficacy evidence of ataluren in patients with nonsense mutation DMD. Neither of the two mentioned trials met its primary endpoint. However, it is known that the sensitivity of the 6MWD across the different ambulatory phases changes. Patients with a baseline 6MWD ≥ 400 m remain relatively stable in physical functioning over 48 weeks, whereas patients with a 6MWD < 300 m are characterized by the most rapid decline and loss of ambulation. Therefore, the grouping of patients by functional status is recommended. Statistically significant differences in 6MDW change from baseline to week 48 between the ataluren-treated group and placebo group were observed in case of all three meta-analyses: the intent-to-treat population, the subgroup characterized by baseline 6MWD ≥300–<400 m (the ambulatory transition phase), and that with 6MWD < 400 m. However, treatment benefit was most evident in patients belonging to the ambulatory transition phase. Statistically significant differences were also observed in some of the secondary endpoints: time to climb four stairs, time to descend four stairs, and time to persistent 10% worsening in 6MWD between the intent-to-treat population and placebo group. In case of the ≥300–<400 m 6MWD subgroup, statistically significant changes favoring ataluren were observed in time taken to walk/run 10 m, climb four stairs, and descend four stairs. In case of the <400 m 6MWD subgroup, statistically significant differences were observed in all secondary endpoints: time to walk/run 10 m, climb four stairs, descend four stairs, and time to persistent 10% 6MWD worsening. Almost all the observed treatment effects were improved compared with those of the individual studies [19]. 

Another open-label extension phase 3 study (NCT02090959) aiming at evaluating the long-term safety data of ataluren in 219 DMD males (aged 7–15 years) was started in March 2014 and ended in June 2018. Patients received ataluren three times a day (10, 10, 20 mg/kg) for up to 144 weeks.

A long-term randomized, double-blind, placebo-controlled study (NCT03179631) followed by a 72-week open-label period was initiated in July 2017. According to the study description around 250 boys aged 7–16 years received ataluren three times a day (10, 10, 20 mg/kg) for 72-weeks. The primary outcome to be assessed is the slope of change in 6MDW over 72 weeks. Its estimated completion date is September 2023. 

In April 2015, a long-term, international, multicenter, observational, post-approval study of Translarna’s safety and effectiveness in usual care was initiated, which was referred to as Strategic Targeting of Registries and International Database of Excellence (STRIDE) (NCT02369731) [55]. The effectiveness of ataluren will be assessed in this ongoing study by comparing results from propensity score-matched patient populations from the STRIDE registry (including patients with nonsense mutation DMD, receiving both ataluren and standard of care) and the Cooperative International Neuromuscular Research Group (CINRG) Duchenne Natural History Study (DNHS) (including patients with any DMD genotype, receiving standard of care only), which is used as a control. Additionally, the link between DMD genotype and ataluren treatment benefit will be assessed using the age at loss of ambulation. Patients will be followed up for at least 5 years from the data of their enrollment. As of the cut-off date of 9 July 2018, 20.2% of patients experienced treatment-emergent adverse events, which were mostly mild or moderate, and in most cases not related to ataluren. The most common adverse effects were gait inability, cough, diarrhea, femur fracture, vomiting, back pain, gastroenteritis, and headache. Few (5.6%) of patients experienced serious adverse events, though all of them were considered as unrelated to ataluren treatment except for left ventricular dysfunction, which was assigned an unknown causal relationship to ataluren. Adverse effects in just 2.3% of patients were considered to be related to ataluren. These included: abdominal pain, diarrhea, vomiting, headache, and increased lipids. A statistically significant difference in age at loss of ambulation in favor of ataluren treatment was observed. The age at the worsening of TFTs (time to stand from supine, time to climb four stairs) was always greater for patients from the STRIDE Registry receiving ataluren. Due to the short duration of the follow-up on ataluren treatment in the STRIDE Registry, conclusions on the cardiac and pulmonary functions could not yet be made. The type of premature stop codon did not influence treatment benefit, indicating that ataluren treatment is not affected by stop codon type. The interim results of the STRIDE Registry described above show that ataluren treatment was well tolerated and provided clinical benefits, including clinically meaningful preservation of ambulation [20,56]. 

The real-life experience of three Italian children with nonsense mutation DMD treated with ataluren (10, 10, and 20 mg/kg) for 1 year was also assessed. The best improvement in 6MWD was observed in case of a patient with a baseline 6MWD of 320 m, who started ataluren therapy at age 5 years. The 6MWD was maintained at a stable level in case of a patient with a baseline 6MWD of 360 m, who started therapy late, at age 10 years. Surprisingly, 50% improvement in 6MWD was observed in case of a patient with baseline 6MWD of less than 75 m, who started therapy at age 8 years. The results of this study indicate that ataluren treatment effectiveness depends not only on disease severity but also the patient’s age at the start of therapy [57]. 

The effect of ataluren treatment in four non-ambulatory nonsense mutation DMD patients (mean age at the start of the treatment 14.1 ± 1.4 years) was also assessed by taking into account cardiac function, pulmonary function tests, and muscle strength. Ataluren was administered orally, three times a day (10, 10, and 20 mg/kg) after mean 4.0 ± 1.0 years of losing ambulation. Serial echocardiography, spirometry, and assessment of muscle strength of period 1 (18–26 months before the start of ataluren treatment to baseline) when compared with those of period 2 (18–26 months after initiation of ataluren) showed mild amelioration of diseases progression after initiation of ataluren treatment. However, none of the changes was statistically significant. The study cohort was small and characterized by substantial intra- and inter-individual variability. No adverse clinical effects were observed. A possible side effect related to the treatment was a reduction in BMI in three out of four patients [58]. 

The response to the treatment with ataluren was also investigated in a 25-year-old symptomatic DMD female carrier with a nonsense mutation in a dystrophin gene. The patient received 2250 mg/day ataluren for 9 months, reporting subjective well-being and strength improvement. In December 2017, the patient discontinued the treatment for 2 months due to surgery of the broken femur. After the resumption of the drug intake in February 2018, the patient reported an improvement in motor skills, including the recovery of walking without support [59]. 

### 2.7. Clinical Studies in Cystic Fibrosis

#### 2.7.1. Cystic Fibrosis

CF is a disabling and life-threatening autosomal recessive disease caused by deletions, missense, and nonsense mutations [33,60]. It is commonly encountered in the Caucasian population [61]. A defective gene identified in 1989 encodes the CFTR protein, which is a novel chloride channel regulated by cAMP [61]. It promotes chloride ions efflux and secondarily inhibits constitutive sodium influx via the epithelial sodium channel [29].

Loss of chloride channel function in the apical membrane of secretory epithelia which line, among others, the respiratory tract, pancreas, small intestine, and male reproductive system results in abnormal water transport and viscous secretion. The affected epithelia are impermeable to chloride ions. As a consequence, the transport of sodium chloride and water across epithelia is disrupted, resulting in altered composition and increased viscosity of epithelial fluids [61]. The variable disease phenotype is clinically described by the level of pancreatic function. Class I mutations of CFTR include premature termination codons or stop codons [62]. The nonsense mutations account for about 10% of cystic fibrosis cases worldwide (>60% of CF patients in Israel), and the most common is the G542X mutation encoding TGA codon [63,64]. Decreased levels of functional CFTR protein in those patients are mainly associated with exocrine pancreatic insufficiency phenotype [62]. Apart from that, progressive respiratory dysfunction occurs in all CF patients with frequent coughing and pulmonary infections, which are the primary cause of morbidity in CF patients [26,65]. To monitor this progression, two spirometry parameters are commonly used: forced vital capacity (FVC), which is the total volume of air (in liters), that can be exhaled during a maximal, forced expiration effort, as well as forced expiratory volume in 1 sec (FEV_1_), which is the volume of air (in liters) exhaled in the first second during forced exhalation after maximal inspiration [66,67]. Healthy individuals usually have FVC and FEV_1_ at least 80% of the predicted values obtained from a normal population [68]. Current medical treatments of CF patients are palliative [29].

#### 2.7.2. Phase 2 and 3 Trials 

The first short-term phase 2 prospective study (NCT00237380) was performed on 24 adults (aged 18 to 56) suffering from CF (characterized by the presence of two disease-causing *CFTR* mutations, with at least one being nonsense) and taking neither systematic nor inhaled aminoglycosides. Patients were administered powder suspension of PTC124 orally three times a day at two dosage regimens (4, 4, 8 mg/kg or 10, 10, 20 mg/kg in the first and second 28-day study cycle, respectively) for 14 days, which was followed by 14 days without treatment. Among the three responsive mutation genotypes studied: 3849 + 10 kb C → T (insertional mutation resulting in an elongated mRNA, containing an in-frame premature UAA stop codon), W1282X (TGA-A), and G542X (TGA-G), the last two were dominant. The functional activity of full-length CFTR protein was assessed by the following endpoints. Increased mean CFTR-mediated total chloride transport (sum of the intrinsic and stimulated chloride transports) was observed in both treatment cycles. Sixteen out of 23 patients in the first cycle and eight out of 21 patients in the second cycle responded to the treatment, which was predefined as an increase in total chloride transport indicated by a change of −5 mV or more. The contributions of intrinsic and stimulated chloride transports were approximately equal. Seven patients responded to the treatment in both cycles. The normalization of chloride transport (hyperpolarization, predefined as nasal potential difference lower or equal to −5 mV) was observed in 13 out of 23 patients in the first cycle of treatment and nine out of 21 in the second cycle, irrespective of the mutant genotype. Observed changes were not dose-dependent but, what is more important, their magnitude was similar to that induced by the topical application of gentamicin to the nasal mucosa and even greater comparing to changes associated with topical gene transfer therapies. In the first treatment cycle, small increases in FEV_1_, FVC, and bodyweight were also observed in most patients. The analysis of circulating neutrophil counts showed a decrease after the treatment phase of the second cycle. There were no changes in lymphocytes or sweat chloride concentration. Several patients reported cough reduction. No serious drug-related side effects were reported, only constipation and mild intermittent dysuria [29]. 

Another phase 2 study (NCT00351078) involved 19 CF adults (aged 19 to 57 years), who participated in the previous short-term study (NCT00237380). They were given ataluren three times a day continuously for 12 weeks at either a lower (4, 4, and 8 mg/kg) or higher dose (10, 10, and 20 mg/kg) within 30 min after a meal. The dosage regimens were assigned to patients based on their response observed in the previous study, in terms of the improvement in total chloride transport. As a result, 12 patients were assigned to lower doses level, as they responded to it better in the previous short-term study, and seven were assigned to the higher doses level for the same reason. Three nonsense mutation genotypes were represented (3849 + 10 kb C → T, G542X, W1282X) with the last one being most common. The predominant premature stop codon was TGA. Five patients had nonsense mutations on both alleles. The chronic ataluren administration used in this study produced a time-dependent statistically significant improvement in total chloride transport with the magnitude of changes irrespective of the dose level. The contributions of intrinsic and stimulated chloride transports were again approximately equal. More than half (61%) of patients responded to the treatment and 56% showed hyperpolarization. Intriguingly, response criteria were not met in the case of the 3849 + 10 kb C → T patient, who had responded in the prior study. There was no significant difference between patients bearing nonsense mutations on just one allele comparing with those bearing them on both alleles. No effect of sex or age on the outcomes was observed. CFTR function increased with time and was associated with improving pulmonary function, although the changes in FEV_1_ and FVC were not statistically significant, but they coincided with the greatest mean improvement in total chloride transport and were reversed upon the end of treatment. Cough was quantified using a mobile, compact, continuous recording system (the VivoMetrics LifeShirt^TM^), and a statistically significant reduction in waking cough frequency was observed in both dosage groups. Furthermore, a significant and time-dependent decrease in cough duration was also observed. No changes in any way low sleeping cough rates were observed. Adverse clinical and laboratory abnormalities (ALT, AST, bilirubin, γ-glutamyltransferase, renal parameters, adrenocorticotropic hormone, and cortisol levels) were uncommon and usually mild (e.g., grade 1 dysuria in 3 patients). No notable new health conditions were reported post-study (≤25 months). This long treatment study documented the persistence of the ataluren pharmacological effect over time, supporting its safety profile [37].

The observed progressive improvements over an extended period of ataluren treatment, in terms of the time-dependent pattern of total chloride transport changes, cannot be explained by pharmacokinetics itself, as the mean ataluren plasma exposures were consistent throughout the study. Possibly, an increasingly greater proportion of epithelial cells expressed CFTR which, together with an increasing amount of CFTR expressed per cell increased the magnitude of chloride transport correction [37]. 

A subsequent multicenter phase 2 study (NCT 00458341) was performed to assess the activity, safety, and pharmacokinetics of ataluren in 30 children (aged 6–18) with at least one nonsense mutation in *CFTR* gene. All three stop mutations were represented by nine nonsense mutations genotypes (Q493X (TAG), G542X(TGA), R553X (TGA), W846X (TGA), W882X (TAG), E1104X (TAA) [69], R1162X(TGA), W1282(TGA) and Q1313X (TAA)), with TGA being predominant. Enrolled patients did not use systematic or inhaled aminoglycosides. The same dosage regimen as in the phase 2 study described above (NCT00237380) was used (two 28-day cycles, with 14 days of treatment and 14 days of follow-up each), except that the order in which dose levels were administered was random. PTC124 induced statistically significant nasal chloride transport response (predefined as at least a −5 mV improvement), which was predominantly due to intrinsic chloride transport changes, in 50% of patients (53% patients in the low-to-high dosing cohort and in 47% patients in the high-to-low dosing cohort). Ataluren treatment resulted in a statistically significant improvement in the proportions of patients with hyperpolarization (predefined as a value more electrically negative than −5 mV) in 47% of patients in either treatment cycle. This outcome was observed to be dose-dependent in both dosage groups. Taking this together, ataluren induced a statistically significant improvement in CFTR function exceeding that observed in pediatric patients with similar genotype receiving systematic gentamicin. Observed changes, in terms of their magnitude and the number of patients responding, were comparable to those in CF adults and were not affected by intrinsic factors such as sex and age. Interestingly, the ataluren-mediated improvements in chloride transport in children seem to be more lasting during off-treatment periods as compared to adults’ treatment. Positive responses to the treatment (either total chloride response or hyperpolarization) were observed in seven out of nine nonsense mutations genotypes in either one or both treatment cycles (except for W846X (TGA-A) and Q1313X (TAA-G)). Among these genotypes, G542X was most responsive to the treatment. However, no changes were observed between homozygous (either G542X/G542X or R1162X/R1162X) and heterozygous (nonsense mutation in only one allele) patients in terms of the improvement in total chloride transport. The proportion of cells expressing apical full-length CFTR protein was also shown to be increased by immunohistochemistry of the epithelial cells obtained from nasal brushings. No frequent or severe adverse effects or laboratory abnormalities (ALT, AST, bilirubin, γ-glutamyltransferase, renal parameters, adrenocorticotropic hormone, and cortisol levels) were observed. A 21–31-month safety follow-up of patients did not show any delayed adverse drug effects. No improvements in FEV_1_, FVC, or body weight were observed. Pharmacokinetic parameters were similar to those observed in adults [38]. 

In 2009, a phase 3 (NCT00803205) international (36 sites at 11 countries in North America and Europe), multicenter, randomized, double-blind, placebo-controlled trial was initiated. Its aim was to evaluate the long-term clinical efficacy and safety of 48-week ataluren therapy at the 10, 10, and 20 mg/kg dose level in 238 patients older than 6 years with at least one CFTR nonsense mutation. The differences between genotype combinations were not clinically significant. The most common nonsense mutations were R553X (18 patients), R1162X (22 patients), G542X (83 patients), and W1282X (86 patients) present in one or both alleles. The primary endpoint of the study was the relative change in percentage of predicted FEV_1_ at week 48. Ataluren resulted in a smaller decrease in FEV_1_, but the difference between ataluren and placebo was not statistically significant. The major secondary clinical endpoint included exacerbation frequency, which turned out not to be statistically different between the treatment and placebo group. Interestingly, statistically significant differences in relative change from baseline in percentage of predicted FEV_1_ (5.7% favoring ataluren) as well as in exacerbations rate (40% lower in the ataluren group) between the treatment and placebo groups were observed when patients using chronic inhaled tobramycin were excluded from analyses. The secondary pharmacodynamic endpoints such as nasal potential difference evaluation of total chloride transport, sweat chloride concentration, chloride transport response, and hyperpolarization were not significantly different between the two groups under investigation. However, the outcomes may be biased by the reproducibility issues of potential difference determination at many different sites and a large number of false positives [33]. Moreover, it is not rare to observe discrepant results in potential difference and sweat chloride concentration endpoints in many clinical trials of CFTR restoration therapies [70]. The reasons may include but are not limited to variable tissue drug availability, CFTR regulation in different cell types, or the responsiveness of mutant CFTR in different tissue compartments. Other endpoints such as cough frequency, inflammation, computerized tomography, weight, and health-related quality of life were not either statistically significant. Safety profiles in both groups were similar except for creatinine elevations occurring in the ataluren group, which were mainly associated with the use of nephrotoxic antibiotics to treat exacerbations and, in some cases, dehydration. Most treatment adverse events were mild or moderate [33].

A more sensitive alternative to FEV_1_ to monitor structural lung changes is chest computerized tomography (CT) [71,72]. However, the decline in FEV_1_ that was observed in both study groups surprisingly was not associated with the disease progression assessed by means of CT scores using the Brody-II score. The reanalysis of all chest CT scans collected in this study using two more sensitive and better-standardized scoring systems (the CF-CT and Perth-Rotterdam Annotated Grid Morphometric Analysis for CF (PRAGMA-CF)) was performed to characterize the progression of structural lung disease over the 48 weeks. Only PRAGMA-CF subscores (percentage of disease mostly due to percentage of mucus plugging) showed progression over 48 weeks. This scoring system has been previously shown to be more sensitive for the assessment of early CF lung disease comparing with the other two mentioned above [73]. This analysis shows that it is also a more sensitive system for more advanced disease stages in older patients. However, no significant differences in the progression of structural lung disease between the two study groups were identified using either scoring system, even with the exclusion of tobramycin-taking patients [74].

The efficacy and safety of ataluren in 279 nonsense-mutation CF patients (older than 6 years) not receiving chronic inhaled aminoglycosides were assessed in international (75 sites in 16 countries in North America, South America, Europe, and Israel), randomized, double-blind, placebo-controlled ataluren confirmatory phase 3 study (NCT02139306), which started in August 2014. Patients with a nonsense mutation in at least one CFTR allele were administered ataluren or placebo three times a day (10, 10, and 20 mg/kg) for 48 weeks. Among the represented genotypes G542X (31% of patients), W1282X (24.5% of patients), R553X (14% of patients), and R1162X (8% of patients) were the most abundant nonsense mutations. The primary endpoint, which was the absolute change in average FEV_1_ from baseline to the average of weeks 40 and 48, was less negative for the ataluren group comparing with the placebo group, but the difference was not statistically significant. The pulmonary exacerbation rate per 48 weeks (lower for ataluren) was not either significantly different. Both study groups experienced modest BMI increases during the study. The differences in BMI and respiratory quality of life during the study between the two groups were not significant, either. No life-threatening adverse events were observed in either group; however, the ataluren-treated group experienced more treatment-emergent adverse events (e.g., gastrointestinal disorders, grade 3 nephrolithiasis). The failure of ataluren therapy to produce clinical benefits in this study can be explained by poor availability of ataluren in the lung, low levels of *CFTR* mRNA available to readthrough (due to NMD or the unfolded protein response), and/or inappropriate amino acid inserted by near-cognate tRNA (resulting in a protein with reduced function or non-functional one) [75]. 

No meaningful clinical benefit was observed in either N-of-1 clinical trial with ataluren and ivacaftor in any combination to treat patients bearing W1282X mutation [76]. 

The summary of clinical trials is presented in Table S1 in the Supplementary Materials. 

### 2.8. Ataluren Readthrough Activity in Other Diseases

#### 2.8.1. Muscles

The loss of the protein dysferlin due to mutations in the *DYSF* genes results in defective muscle membrane repair leading to muscle breakdown, which is the basis of either limb-girdle muscular dystrophy type 2b (LGMD2B) or Miyoshi myopathy [77]. Among the nonsense mutations in the *DYSF* gene, R1905X is prevalent in specific populations [78]. Dysferlin was shown to be crucial for membrane blebbing in skeletal muscle myotubes in response to hypotonic shock using two new in vitro assays with dysferlin-deficient mice and human myocytes. PTC124 at the concentration of 10 μg/mL was shown to induce readthrough of the premature stop codon in human myocytes obtained from a Miyoshi patient with an R1905X mutation. The level of full-length protein expressed (approximately 15% of the normal level determined by Western blot) was high enough to rescue the myotube membrane blebbing indicating potential therapeutic use of ataluren in dysferlin-deficient patients harboring nonsense mutations [79]. 

#### 2.8.2. Heart

The human ether-a-go-go-related gene (*HERG*) encodes the α subunit of the rapidly activating delayed rectifier potassium current channel (*I*_Kr_) in the heart, which is important for cardiac repolarization. *I*_Kr_ dysfunction may lead to type 2 long QT syndrome (LQTs) [80]. LQTs is an inherited cardiac arrhythmia syndrome that can result in sudden death [81]. Mutations in 13 genes encoding ion channels or structural protein are associated with LQTs [80]. Type 2 LQTs is caused by mutations in *HERG*, among which 10% are nonsense mutations [82]. In vitro experiments with HEK293 cells followed by Western blot analyses showed a significant dose-dependent increase in the full-length protein expression due to R1014X readthrough by ataluren (up to 10.3% of the wild type for the concentration of 56.8 μg/mL, 24 h incubation time). Further experiments with other nonsense mutations showed a slight but significant increase in full-length protein expression level in case of W927X mutations, but not in case of R863X and E698X, suggesting that as the mutation site approaches the N-terminal, the rescue efficiency decreases. The functionality of the expressed protein was demonstrated by patch-clamp recording. Ataluren (56.8 μg/mL) demonstrated a slight but significant increase in the peak tail current densities of R1014X channels at 40 mV. However, the drug was not able to correct the leftward shift of the activation curve [80]. 

#### 2.8.3. Eyes/Vision

The efficacy and safety of ataluren were tested on two choroideremia (CHM) model systems [30]. CHM is an X-linked recessive inherited disease involving the degeneration of the retinal pigment epithelium and the neighboring choroid and retinal photoreceptor cell layers, leading to blindness [83]. Over 30% of CHM patients bear a nonsense mutation in the *CHM* gene [84], encoding 653 amino acid protein, Rab Escort Protein 1 (REP1), which is involved in the prenylation of Rab proteins. The binding of hydrophobic motifs at their C termini facilitates their intracellular membrane transport trafficking [85]. 

PTC124 treatment of chm^ru848^ zebrafish embryos, harboring a TAA nonsense mutation, led to a two-fold increase in survival, prevented the onset of retinal degradation with reduced oxidative stress and apoptosis, increased REP1 protein by 23.1%, and restored biochemical function as evidenced by in vitro prenylation assays. Quantitative RT-PCR experiments showed higher levels of transcripts in ataluren-treated chm^ru848^ zebrafish. Additionally, the renal function and ototoxicity of ataluren in this model were assessed. At the concentration used in all the experiments (2.8 μg/mL no signs of renal or ototoxicity were observed. The chm^ru848^ mutants treated with different PTC124 doses showed a bell-shaped curve, with reduced survival at 1.4 and 4.2 μg/mL, compared to untreated embryos. 

Using the other model, human primary fibroblast cell line, CHM^Y42X/y^, bearing TAG mutation, recovered prenylation activity was also shown, indicating functional full-length protein formation. However, no detectable increase in REP1 protein or its transcript level was seen in these cells at 11.4 μg/mL ataluren concentration [30]. 

Ataluren was also used to promote the readthrough of nonsense mutations in the *RP2* gene, which lead to a severe form of X-linked retinitis pigmentosa. The most common *RP2* mutation is R120X. The RP2 protein seems to be implicated in cilia-associated traffic of some proteins e.g., Gβ1. The R120X patient cells showed no signs of full-length or truncated RP2 protein expression in immunocytochemistry and Western blot analyses. Furthermore, they were characterized by the mislocalization of IFT20 and Gβ1 and a disrupted Golgi morphology, which are cellular phenotypes characteristic of the lack of RP2 function. Fibroblasts derived from an *RP2* patient carrying the nonsense mutation R120X were reprogrammed into induced pluripotent cells (iPSC) and differentiated into retinal pigment epithelial cells. A single dose of ataluren (2.8 μg/mL) was able to restore the expression of endogenous, full-length, functional RP2 protein up to 13% of control cells in the R120X fibroblasts, with no effect on the mRNA level. Additionally, ataluren treatment was able to restore the RP2-null cellular phenotype in terms of IFT20 and Gβ1 correct localization as well as correct Golgi morphology in both R120X fibroblasts and R120X iPSC-derived RPC cells. Thus, translation readthrough therapy could be beneficial for patients suffering from *RP2* nonsense mutations [86]. 

Another study investigating inherited retinal dystrophies used fibroblast-derived induced pluripotent stem cells to generate retinal pigment epithelium (RPE) obtained from an individual suffering from retinitis pigmentosa caused by a nonsense mutation in the *MERKT* gene. MER receptor tyrosine kinase (MERTK) is expressed at the apical surface of the RPE and plays an important role in phagocytosis by RPE cells [87]. PTC124 (10 μg/mL) was shown to restore the expression of MERTK protein and RPE phagocytic activity (12% of control RPE level), which suggests that ataluren can be used to treat retinitis pigmentosa due to nonsense variants in MERTK, which has no effective therapies so far [88]. 

The potential of PTC124 treatment to readthrough premature stop codons was also evaluated using the retinitis pigmentosa GTPase regulator (*RPGR*) gene [89]. Mutations in the *RPGR* gene are the most common causes of X-linked retinitis pigmentosa (RP), which is characterized by an early onset and a rapid progression of the visual defects [90]. Up to 20% of the reported mutations in *RPGR* are nonsense mutations, being the second most common mutation type. The higher efficacy of the ataluren readthrough was shown for UGA as compared to UAA using expression constructs in HEK293T cells and 8.5 μg/mL ataluren concentration. PTC124 was also able to partially restore RPGR expression (8% treatment efficacy) and its localization at the primary cilium in a patient-derived fibroblast cell line with a hemizygous nonsense mutation (L385X), showing a promising therapeutic approach [89]. 

Pseudoxanthoma elasticum (PXE) is an autosomal recessive disorder characterized by ectopic connective tissue mineralization potentially resulting in serious complications in the eyes and the cardiovascular system such as loss of visual acuity, hypertension, and occasional strokes [91]. It is caused by mutations in the *ABCC6* gene, 35% of which are premature nonsense mutations, resulting in the synthesis of truncated, non-functional ABCC6 protein, being a transmembrane efflux transporter expressed primarily in the liver and kidneys [92]. The most common mutation is R1141X (TGA-A) [93].

The incubation of HEK293 cells transfected with human *ABCC6* expression constructs harboring seven different PXE-associated mutations (shown in Table 1) with ataluren (1.2, 2.5, 5, 10, 20, and 40 μg/mL) up to 72 h resulted in the expression of full-length ABCC6 polypeptide. Immunofluorescence and In-Cell ELISA analyses demonstrated the highest level of expression in case of R1275X and R1164X mutations (R1275X > R1164X > R1141X > R378X > Q1143X > Q518X > R1398X) and 5 μg/mL ataluren concentration. The readthrough efficiency was not only dose- and sequence context-dependent but also increased with longer incubation time. Ataluren showed no cytotoxicity in concentration up to 20 μg/mL. Furthermore, the functionality of the full-length polypeptide expressed in the presence of PTC124 was tested using a zebrafish mRNA rescue assay. Mutant human *ABCC6* mRNAs encoding either cysteine, tryptophan, or glycine in position 1141 were injected into zebrafish embryos in parallel to wild-type mRNA encoding wild-type arginine in the corresponding position. All transcripts were able to rescue the *abcc6a* phenotype of zebrafish decreasing lethality, which confirms the functionality of all three protein variants [93].

The human Usher syndrome (USH) is an autosomal recessive disorder caused by mutations in specific genes and characterized by sensorineural hearing loss and retinitis pigmentosa [94]. In-frame nonsense mutations represent around 20% of all identified USH-causing mutations [15]. Among the three clinical subtypes (USH1, USH2, USH3), Usher syndrome type 1 is the most severe form [94]. The *USH1C* gene encodes the scaffold protein harmonin in photoreceptor cells [95,96]. Three nonsense mutations in the *USH1C* gene were identified with R31X containing premature TGA stop codon. The R31X mutation results in a truncated harmonin and as a consequence disruption of the USH protein network in photoreceptor cells, leading to retinal degeneration [96]. 

The readthrough efficacy of PTC124 (5 and 10 μg/mL) was first demonstrated using HEK293T cells transfected with cDNAs encoding two mutated harmonin isoforms (a1 and b3). A dose-dependent increase in harmonin expression was observed by immunofluorescence and Western blot in cell cultures media containing PTC124 (2.5% of the wild-type). Low expression was also observed in control cells demonstrating spontaneous readthrough of the R31X nonsense mutation. The expressed proteins were shown to be functional, having scaffold and actin filament bundling properties most probably sufficient for proper retinal function [14]. However, no synergistic effect of co-administration of PTC124 and another readthrough agent, designer aminoglycoside, NB54 was observed [15].

PTC124 treatment of murine retinal explants with introduced harmonin nonsense mutation resulted in increased levels of readthrough as compared with controls that showed some spontaneous readthrough level. The effect of PTC124 (10 μg/mL) was higher than that observed with gentamycin (8.0-fold vs. 3.4-fold, respectively). As previous studies with gentamicin showed that induced readthrough of a nonsense mutation in rhodopsin was enough to slightly improve retinal function, the observed two times greater efficiency of PTC124 suggests that PTC124 treatment should be efficient enough to combat retinal degeneration in USH1 patients. Importantly, PTC124 (5 and 10 μg/mL) was shown to be retinal compatible with no effect on the number of photoreceptor cells or structural integrity of the neuronal retina, which makes it advantageous over gentamicin [14]. No signs of reactivation of evolutionary turned-off pseudogenes were found [15].

Harmonin nonsense mutation was also introduced into the retina of newborn mice in vivo. After subretinal injections of PTC124 (2.5 μg), fluorescence and Western blot analyses of different regions of photoreceptor cells of ex vivo eyes were conducted. Increased full-length protein expression with its proper localization was observed in PTC124-treated mouse eyes [14]. The efficiency of PTC124 to induce readthrough was nearly the same as that of NB54 [15].

Most (55–90%) cases of USH2 are due to mutations in the *USH2A* gene, among which around 16% are nonsense mutations. This gene encodes two isoforms: short USH2A protein and long USH2A called Usherin. They are essential for the maintenance of photoreceptor cells and the normal development of cochlear hair cells. The capability of ataluren to induce translational readthrough of the disease-causing nonsense mutation G3142X in the *USH2A* gene was demonstrated using transfected HEK293T cells and patient-derived fibroblasts. Indirect immunofluorescence staining and Western blot analyses of ataluren treated (10 μg/mL) HEK193T cells showed the full-length protein expression. Expression was significantly greater after treatment with both ataluren and gentamicin (5.7-fold vs. 68.9-fold, respectively). Due to the lack of experimental tests evaluating the functionality of the USH2A protein, in silico approaches were used showing intolerance toward the inclusion of any of the possible amino acids (arginine, leucine, serine, tryptophan, cysteine, glycine). However, Western blot analyses of ataluren-treated USH2A G3142X-patient-derived fibroblasts demonstrated a significant increase in USH2A protein expression following the 5 μg/mL concentration treatment, which was 4.3-fold greater as compared with control, corresponding to approximately 50% protein restoration. Only a weak increase in expression was observed in case of 10 μg/mL ataluren and gentamicin treatments (1.9-fold and 1.3-fold, respectively). Furthermore, indirect immunofluorescence microscopy showed proper membrane protein localization. As Usher syndrome can be considered a ciliopathy, the number of ciliated cells in USH2A patient-derived fibroblasts was compared with that of healthy control fibroblasts using immunofluorescence. Ataluren treatment (5 μg/mL) resulted in an increased number of ciliated cells (from 54% for untreated patients cells up to 78% after the treatment) despite a low level of rescued protein expression, while there was no increase due to gentamicin treatment [97].

Aniridia is a congenital and progressive panocular condition with poor visual prognosis characterized by absence of iris tissue, corneal opacity, glaucoma, cataract, and foveal hypoplasia as well as brain, olfactory, and pancreatic abnormalities [98]. The disease is caused by mutations, among which 50% are in-frame nonsense mutations resulting in paired box 6 (PAX6) (aniridia type II) protein haploinsufficiency. A *Pax6*-deficient mouse model of aniridia, with naturally occurring G194X stop mutation (TGA) in the mouse *Pax6* gene, was used for in vivo studies. Daily subcutaneous injection of 30 μg/mL ataluren from day 4 to 14 after birth resulted in remarkable normalization of the eye malformation defects, including correction of retinal infolding and in an increase in lens size to 70% of that observed in wild-type subjects. However, there was no significant improvement in the thickness of corneal epithelium even after continued ataluren treatment to 60 days after birth, suggesting a limited corneal distribution of the drug from systematic delivery. Furthermore, electroretinography (ERG) demonstrated substantial ERG responses in mice treated with ataluren up to 60 days after birth. Interestingly, withdrawal experiments showed that the effect remained until day 60 after birth upon the cessation of ataluren treatment on day 21 after birth. Additional experiments were performed with 1% aqueous ataluren suspension delivered topically to the eye (50–100 μg per eye two times a day from day 14 to 60 after birth) aiming at increasing drug concentration in the eye, while limiting any systematic toxicity. However, only partial histological rescue of the retina and lens defects were observed with concomitant ocular irritation. Reformulation experiments led to the development of START suspension (0.9% sodium chloride, 1% Tween 80, 1% powdered ataluren, 1% carboxymethylcellulose) with improved particle dispersion properties and increased viscosity. Its application resulted in no irritation and, more importantly, it was able to reverse the lens and retinal defects. Additionally, increased thickness of corneal epithelium reaching levels observed in wild type was observed. ELISA analysis of treated mouse retinal and corneal epithelia protein lysates showed increased PAX6 protein levels (up to 90% of the wild type). START therapy resulted also in statistically significant benefit in terms of EGR testing over systematic and 1% aqueous suspension treatment. Functional effects of the treatment were further evaluated in terms of visual acuity, which was assessed by measuring the optokinetic tracking response mediated through retina–brain circuitry. Again, the best results were shown due to START treatment. The suppression of nonsense mutations by ataluren inhibited disease progression, additionally reversing corneal, lens, and retinal malformation defects and restoring the electrical and behavioral responses of the retina [99].

As neither the chemical stability over time nor sterility of START formulation were assessed, another study reported the development of a 1% ataluren oily solution free of particles that was chemically and microbiologically stable at least over 2 months when stored at 25 °C. These eye drops containing 1% ataluren (m/v) were prepared using DMSO (10%; *v*/*v*) as co-solvent in castor oil (90%; *v*/*v*) and subsequently filtered through a 0.22 μm polyethersulfone filter before being sterilely distributed into the eyedrop low-density polyethylene vials with an innovative insert that maintains sterility after opening. During the study period, no visual changes in color or limpidity were observed. There were no signs of any particulate matter. Eye drops showed chemical stability for up to 60 days, retaining at least 99% of ataluren initial concentration, while STAR ataluren suspension showed greater than 10% loss of ataluren concentration at day 21. The European Pharmacopeia sterility assay showed the absence of bacterial or fungal contamination over 14 days, indicating no necessity to use preservative agents. It was also determined that the oxidation of ataluren required higher hydrogen peroxide concentrations (e.g., 15%). No degradation occurred in the direct photolytic stress condition. However, photocatalyst agent (titanium oxide) induced a rapid degradation of ataluren [100]. 

#### 2.8.4. Respiratory Tract

Heritable pulmonary arterial hypertension (HPAH) is a serious lung vascular disease caused, among others, by heterozygous mutations in the bone morphogenic protein (BMP) pathway genes, *BMPR2* and *SMAD9*, encoding proteins taking part in signaling pathways regulating growth, differentiation, and development. More than one-quarter (29%) of all HPAH mutations are nonsense mutations [101]. Under physiological conditions, type II BMP receptor–ligand binding stimulates the phosphorylation of Smads, which transduces the signal from the cytoplasm to the nucleus, regulating the transcription of the target genes including the family of inhibitors of DNA binding (Ids 1–4) [102,103]. Furthermore, BMP mediates post-transcriptional up-regulation of a subset of microRNAs, amongst which two have growth-suppressive properties in pulmonary vascular cells. The proliferation of endothelial and smooth muscle cells observed in HPAH progressively obliterates the pulmonary arterioles, leading to sustained elevation of pulmonary artery pressure and consequently to heart failure. Several lung- and blood-derived cells of HPAH patients bearing either *BMPR2* (W9X (TGA-C), R213X (TGA-T), R321X (TGA-G), R332X (TGA-G), Q433X (TAG-A), and (E845X (TAA-A)) or *SMAD9* (R294X (TGA-A)) nonsense mutations were analyzed. Ataluren (0.9–6 μg/mL) showed a significant dose-dependent increase in BMP-mediated induction of microRNA processing in six out of seven cases (R332X did not respond), irrespective of a variable and sometimes low mRNA level. The concentration of 4 μg/mL was high enough to achieve complete correction of the fold change in comparison to control cells in case of R321X nonsense mutation. Consistent with other data published, ataluren did not affect the level of *BMPR2* or *SMAD9* transcript nor the amount of NMD. Western blot analyses of endothelial cell lysates treated with 5.7 μg/mL ataluren showed 1.9- and 3.7-fold increase in BMPR2 protein levels in case of R321X and W9X mutations tested. Furthermore, the ligand-dependent phosphorylation of downstream target, Smads, was also increased. Ataluren (5.7 μg/mL) was also shown to reverse the usually hyperproliferative phenotype of pulmonary artery endothelial and smooth muscle cells, as shown with mutations R294X and R321X. Altogether, these results show that ataluren can effectively suppress a high proportion of *BMPR2* and *SMAD9* nonsense mutations and correct BMP signaling in vitro [101]. In another study, ataluren treatment (28.4 μg/mL) of blood outgrowth endothelial cells isolated from a patient harboring the R584X mutation resulted in partial restoration of BMPR2 protein expression. The R584X mutation was characterized by the greatest level of a mutant transcript. PTC124 treatment was also shown to rescue a downstream bone morphogenetic protein signaling target, Id1, reverse increased lipopolysaccharide-induced permeability in mutant cells, and reduce the increased proliferation and apoptosis of R584X blood outgrowth endothelial cells. Importantly, increased BMPR2 protein expression was demonstrated in the lungs of mice bearing the R584X mutation after oral ataluren administration (0.3% PTC124 over two weeks) with no effect on mRNA level. Furthermore, reduced proliferation of pulmonary artery smooth muscle cells isolated from the R584X mutant mouse model was also observed. The collected results show that ataluren could provide beneficial therapy to a specific subset of PAH patients, in whom NMD is less effective [103].

Ataluren was also tested as a potential readthrough therapeutic agent that could be used to treat primary ciliary dyskinesia (PCD) [104]. PCD symptoms include, among others, recurrent and chronic upper and lower airway infections. This rare, predominantly recessive disease is caused by mutations in genes encoding essential structural proteins of cilia [105] or proteins involved in cilia assembly [106]. Since the majority of PCD-causing mutations are nonsense mutations, ataluren’s PTC-readthrough-stimulating efficiency was tested using HEK293 cells transfected with dual-luciferase reporter vectors containing one of six PTC mutations (TGA-C, TGA-C, TGA-A, TGA-T, TAG-T, and TAA-C). Additionally, tests on primary nasal epithelial cells of healthy donors were performed to assess ataluren’s toxicity toward ciliated cells (cell viability and motility of cilia). Ataluren’s concentrations of 2.5 μg/mL, 5 μg/mL, and 10 μg/mL did not negatively impact epithelial airway cells’ viability, as shown in cytotoxicity tests. Cilia motility analyses showed a slight decrease in the ciliary beat frequency due to ataluren treatment. However, the observed changes were within the expected normal range. PTC-readthrough efficiency was assessed by determining the ratio of Fluc (reflecting the presence of the full-length protein) and Rluc (reflecting the presence of the short protein) luminescence in the whole-cell lysates of the ataluren-treated HEK293 cells. In most cases, increases were small and insignificant, which was probably due to cell specificity reasons. However, in case of TAA-C, small but significant increases over the untreated cells were observed for 2.5 μg/mL and 5 μg/mL ataluren concentrations. What must be emphasized is that the observed stimulation potentials detected for gentamicin and G418 in this study were also lower as compared to previously determined. The differences observed may be caused by distortions of the fluc/rluc assay [104]. 

#### 2.8.5. Metabolic Disorders

Neuronal ceroid lipofuscinosis (NCL or Batten disease) is composed of a group of autosomal recessive neurodegenerative lysosomal storage disorders affecting mostly children [107]. Over 400 genetic mutations in at least 14 genes lead to NCL [11]. Mutations in the *CLN1* gene encoding the palmitoyl-protein thioesterase-1 (PPT1) are the basis of infantile neuronal ceroid lipofuscinosis (INCL or CLN1) [108]. PPT1 deficiency causes an abnormal accumulation of palmitoylated proteins being ceroid components. Mutation-affected cells are characterized by the presence of granular osmiophilic deposits (GRODs). Nearly one-third (31%) of patients with INCL in the United States have PPT1 nonsense mutations in the corresponding gene [109]. Mutations in *CLN2* encoding tripeptidyl-peptidase 1 (TPP1) are the basis of late-infantile neuronal ceroid lipofuscinosis (LINCL or CLN2) [108]. Around 16% of all *CLN2* mutations are nonsense mutations [110]. Another type of NCL, juvenile NCL (CLN3), is caused by loss-of-function mutations in the *CLN3* gene encoding a transmembrane endosomal/lysosomal protein, CLN3, or battenin [111]. 

PTC124 treatment (0.15–15 μg/mL) of cultured INCL patient-derived cells carrying a nonsense mutation induced dose- and time-dependent PPT1 modest enzymatic activity (1–1.3% of normal), which was comparable with that induced by gentamicin treatment, with no adverse effects on cell viability. Interestingly, the effect increased up to the drug concentration around 3 μg/mL, reaching a plateau and then decreasing at around 7 μg/mL. Despite the rather low enzymatic activity observed, beneficial biological effects in INCL patient-derived lymphoblasts were demonstrated in terms of the reduction of ceroid load. Additionally, PTC124 treated cells were shown to contain appreciably less GRODs comparing with the untreated cells. Furthermore, ataluren reduced INCL lymphoblasts apoptosis, which is increased in INCL patients. A possible limitation of ataluren’s treatment is its ability to cross the blood–brain barrier, which should not be the problem in the early stages of the treatment when this barrier is most likely damaged. However, the hydrophobic character of PTC124 should allow it to cross the treatment-restored impermeability of the blood–brain barrier [109].

Treatment of INCL and LINCL lymphoblast cell lines (*CLN1* R151X/R151X and *CLN2* R208X/L104X) with ataluren (0.625–5 μg/mL) for 48 h resulted in increased PPT1 (up to 4% of normal activity) and TPP1 (up to 8% of normal activity) enzyme activity, respectively. However, the observed increases were significant only at ataluren concentrations of 2.5 and 5.0 μg/mL. The observed increase in PPT1 and TPP1 enzyme activity can alleviate INCL pathogenesis [11]. 

Nonsense suppression by PTC124 was also shown using human neuronal cell models of late-infantile NCL and juvenile NCL. Incubation with the drug at the concentration of 5.1 μg/mL resulted in both a small, but significant increase in TPP1 activity and the attenuation of neuropathy in NCL patient neuronal progenitor cells derived from induced pluripotent stem cells (iPSCs) [111].

Another lysosomal storage disorder caused, among others, by nonsense mutations is mucopolysaccharidosis type VI (MPS VI). The resulting arylsulfatase B (ARSB) deficiency leads to widespread intra- and extra-cellular accumulation of glycosaminoglycans and affects different organs such as the eyes, heart, bones, and joints [112]. The readthrough efficacy of ataluren (1 μg/mL and 2.8 μg/mL) to induce the full-length functional ARSB protein expression was tested using fibroblast cell lines of patients carrying four ARSB nonsense mutations (R315X (TGA-G), R327X (TGA-G), Q456X (TAA-T), and Q503X (TAG-T)). In all cases except one (Q503X), ARSB activity was increased. The greatest, and more importantly statistically significant effect was observed in case of R327X, which was twice and thrice that of untreated cells for ataluren doses 1 μg/mL and 2.8 μg/mL, respectively. For R315X and Q456X mutations, only higher ataluren-dose treatment resulted in an ARSB activity increase (twice that of untreated cells); however, the changes were significant only in case of R315X mutation. Statistically significant increases reaching ≤2.5% of the wild-type fibroblasts were high enough to significantly reduce the lysosomal size, suggesting that PTC124 induced clearance of lysosomal storage. A notable advantage of ataluren as a therapeutic agent in this disease is its low molecular weight, making it more diffusible than conventionally used proteins, thus easier reaching disease sites such as bones or cartilages [113].

Carnitine palmitoyltransferase 1A (CP1A) deficiency is an autosomal recessive disorder of hepatic mitochondrial long-chain fatty acid oxidation. It is caused by various mutations, including nonsense ones resulting in the expression of a truncated protein, which is predicted by protein modeling to lack the catalytic core and the carnitine-binding motif [114]. In most CPT1A patients, residual enzyme activity is reduced to less than 5% of normal [115]. The efficiency of ataluren treatment (1.4 μg/mL) was tested using skin fibroblasts of a CPT1A-deficient patient with the homozygous mutation R160X. The *CPT1A* mRNA level was not affected by ataluren treatment remaining at a 10-fold lower level comparing to the normal control cells, while low levels of full-length CPT1A protein were detected by Western blot. Furthermore, enzyme activity increased to 45% of control, whilst gentamicin treatment resulted in just a 36% increase. Intriguingly, the normal CPT2 activity also increased on PTC124 treatment in patients’ cells, but not in normal cells, which may be due to the activation of the CPT2 enzyme with the onset of fatty acid flux resulting from the presence of functional CPT1A [116].

Methylmalonic aciduria (MMA) is a rare inherited autosomal recessive disorder caused either by a defect in the methylmalonyl-CoA mutase enzyme or in the biosynthesis of adenosylcobalamin cofactor [117]. It is characterized by a build-up of the metabolite methylmalonic acid. Around 16% of methylmalonic aciduria mutations are caused by nonsense mutations [118]. The ability of ataluren to readthrough the stop codon within the *MUT* gene was shown in BAC_MMA*EGFP genomic reporter assays, which is a HeLa cell line with a bacterial artificial chromosome construct containing the R403 stop mutation and enhanced green fluorescent protein in-frame of the human methylmalonyl-CoA mutase locus (*MUT*). Treatment with 5.7 μg/mL ataluren resulted in an increased reporter protein mRNA expression (2-fold at 72 h incubation). Flow cytometry confirmed increased reporter protein expression. However, there was only a slight 33% statistically insignificant increase in MMA enzyme activity. Furthermore, mouse fibroblast cells, carrying a stop-codon mutation, treated with either ataluren or gentamycin showed an increase in mRNA level (1.6-fold and 1.5-2-fold, respectively) and methylmalonyl-CoA mutase enzyme activity (25% and 32%, respectively). However, the increase due to ataluren was not statistically significant [31].

Propionic acidemia is the autosomal recessive disease caused by mutations in the *PCCA* and *PCCB* genes, including nonsense mutations representing 8 and 11% of the mutations in the *PCCA* and *PCCB* genes, respectively. Most of them contain TGA stop codons [119]. The *PCCA* and *PCCB* genes encode both subunits of the biotin-dependent propionyl-CoA carboxylase enzyme (PCC), which catalyzes the conversion of propionyl-CoA to methylmalonyl-CoA. In vitro translation experiments and in silico analyses using *PCCA* sequences with introduced R313X (TGA-A), Q371X (TAG-G), Y380X (TAA-C), S562X (TGA-G), and Q611X (TAG-A) nonsense mutations as well as those using *PCCB* sequences with G94X (TGA-A), R111X (TGA-G), R113X (TGA-G), Q139X (TAA-A), Y314X (TAA-A), R514X (TGA-A), and W531X (TGA-A) nonsense mutations were performed to test the functional effect of the predicted changes in amino acid identity inserted at the premature stop codon. According to expression analysis, the translation of most nonsense mutations should result in original amino acid incorporation or a missense mutation with partial enzyme function. However, in silico predictions indicated a rather damaging effect for most of the missense changes, producing conflicting results in some cases. Fibroblasts obtained from patients P1 and P2, carrying R313X and S562X homozygous mutations, respectively as well as from patients P3, P4, and P5 characterized by the following heterozygous mutations: W531X/G470fs, Y314X/E331X, and G94X/G470fs, respectively, were treated with ataluren at two different concentration levels, 2.8 and 5.6 μg/mL. Detectable but very small increases in the PCC activity were observed in P1 (lower dose: 0.5%, higher dose: 0.2%), P3 (only for lower dose: 1%), and P5 (lower dose: 0.7%, higher dose: 1.2%). A little bit higher values were obtained in case of P2 (lower dose: 0.6%, higher dose: 2.5%) and P4 (lower dose: 0.2%, higher dose: 6.7%). The P2 patient was homozygous for nonsense mutations, predictably resulting in a protein with high residual activity. Surprisingly, P3 with a heterozygous nonsense mutation predictably reverting to the original amino acid showed only low activity. mRNA levels after ataluren treatment were not significantly changed in P1–P4 fibroblasts except for P2 [119]. 

#### 2.8.6. Neurological Disorders

Four-day ataluren treatment (2.8 and 8.5 μg/mL) of a lymphoblastoid cell line derived from ataxia-telangiectasia patients with AT153LA (TGA-A), homozygous *ATM* nonsense mutation resulted in an increase in the ATM kinase activity. The observed increase was greater in case of the lower dose. However, none of the increases was significant [120].

#### 2.8.7. Kidney

Ataluren was administered intraperitoneally (60 mg/kg) from postnatal day 6 to a mice model of isolated proximal renal tubular acidosis [121]. This condition can be caused by mutations in the basolateral electrogenic Na^+^/HCO_3_^−^ cotransporter (NBCe1) gene (*SLC4A4*) [122]. The presence of nonsense mutations in *SLC4A4* can also result in growth and mental retardation, corneal opacities, glaucoma, cataracts, and basal ganglia calcification [123]. An animal model of the disease was created—NBCe1 W516X knock-in mice, lacking functional and morphological NBCe1 protein and manifesting early lethality. PTC124 treatment significantly increased the survival rate, blood pH, and bicarbonate levels and decreased plasma creatinine and blood urea nitrogen concentrations. Importantly, it also resulted in significantly greater expression of full-length NBCe1 protein, with no alteration of mRNA level, and partially rescued NBCe1 activity ex vivo [121]. 

### 2.9. Ataluren in Polytherapy 

The effect of the combination of PTC124 and amlexanox (NMD inhibitor) was evaluated quantitatively by measuring iodine efflux across plasma membrane using 6CFSMEo-cells. Ataluren showed a significant increase in the export of iodine (especially at the concentration of 7.1 μg/mL), and its combination with amlexanox seemed to be slightly more sufficient than each molecule alone [124].

### 2.10. Limitations of Readthrough Assays

In luciferase cell-based translational assay, the firefly luciferase (FLuc) gene containing a premature stop codon (TGA) at position 190 is inserted into HEK293 cells. The level of luciferase-mediated chemiluminescence correlates directly with the extent of translational readthrough at the site of nonsense mutation [33]. Cell-based FLuc reporter assays lead to the identification of PTC124 as having nonsense codon suppression activity [23]. However, PTC124 was shown to be a highly potent Fluc inhibitor [125]. What is more, assays with a different luciferase reporter (*Renilla reniformis* luciferase, Rluc) containing a TGA stop codon at position 110 did not confirm ataluren’s readthrough activity. That is why it was suggested that the initially observed up-regulated FLuc activity due to ataluren treatment may be an artifact resulting from the direct reversible interaction between ataluren and a small amount of full-length Fluc protein produced due to natural readthrough of the TGA. The accumulation of stabilized luciferase results in an increase in signal measured when the excess reporter substrate added effectively competes off a stabilizing inhibitor. To confirm this mechanism, an additional experiment was performed, in which ataluren at a concentration of 0.57 μg/mL was shown to protect purified FLuc from trypsin digestion, increasing its half-life. These results indicate that ataluren induces such conformation of luciferase that is less susceptible to proteolysis, consequently increasing its effective concentration [125]. Further X-ray crystallography studies revealed that the stabilization results from the formation of an inhibitory product formed during the FLuc-catalyzed reaction between its natural substrate, ATP, and PTC124, namely the acyl-AMP mixed-anhydride adduct, PTC124-AMP. The inhibitory activity of this multisubstrate adduct inhibitor is relieved by free coenzyme A, which is an abundant component of luciferase detection reagents used in cell-based assays. That is why PTC124 appears to increase instead of inhibiting Fluc activity in these assays, and the luminescent signal produced was mistakenly attributed to the readthrough ability of PCT124 [126,127].

A diverse range of in vitro stably or transiently transfected cell-based reporter assays (Table 2) was conducted, confirming either ataluren’s off-target Fluc activity in some assays or showing no activity in other assays. 

Using the stably transfected Fluc cell-based reporter assay, it was shown that at concentrations greater than 0.043 μg/mL, ataluren inhibits Fluc activity in a dose-dependent manner. A possible reason is the continued presence of the compound inhibiting the enzymatic activity of the assay protein when the detection buffer is added to the cells. This phenomenon could result in masking a more pronounced stimulation of Fluc activity due to readthrough. However, when the compound was removed from cells by washings with media before the addition of detection buffer, no significant increase in signal was observed, which confirms a lack of readthrough activity. These observations were supported by further experiments using stably transfected AD293 cells with a wild-type Fluc2P construct in which the behavior of ataluren was identical to the one observed with the construct containing an in-frame nonsense mutation. These observations confirm that PTC124 increases the FLuc signal by stabilizing the protein being a product of natural readthrough from proteolytic degradation. No signs of readthrough activity were observed due to ataluren treatment using other reporter assays characterized by different levels of sensitivity. However, the limitation of these experiments is that all of them used cDNA constructs, which are not subjected to mRNA regulatory processes. What is more, the in vivo activity of PTC124 may be different than its in vitro/ex vivo [128].

The ability of PTC124 to promote readthrough was experimentally tested using a new reporter assay, being orthogonal to previously used enzymatic tests. The reporter vector was based on a plasmid bearing the H2B histone coding sequence fused in frame with the green fluorescent protein (GFP) cDNA. A TGA stop codon was introduced into the H2B-GFP gene using site-directed mutagenesis. Fluorescence was not detectable upon G418 or PTC124 treatment, suggesting that a small amount of protein was expressed as a result of the readthrough. However, the expression of the H2B-GFP fusion protein was monitored on a cell base by fluorescence microscopy on live cells. PTC124 at concentrations of 1.7, 2.6, and 3.4 μg/mL induced the dose-dependent production of full-length functional H2B-GFP protein, despite the presence of a premature stop codon. Additionally, the computational study showed a specific interaction between ataluren and the UGA codon (G542X) found in the 11-codon sequence corresponding to a CFTR mRNA fragment. Both these results confirm that PTC124 is able to promote specific readthrough on internal UGA premature stop codons [129]. 

### 2.11. Factors Determining the Response to Nonsense Suppression Therapy with Ataluren

The readthrough efficiency may be affected by the premature stop codon identity, its context [29] as well as mutation position relative to the N-terminal end [80]. However, the data published are inconclusive. In the preclinical tests, it was shown that ribosomal readthrough of UGA-G (G542X mutation) induced by PTC124 was more efficient than that in case of UGA-A (W1282X mutation) [23]. On the other hand, in case of HEK293 cells transfected with the human *ABCC6* expression construct, the highest level of expression was observed in case of UGA-C and the lowest was observed for UGA-G [93]. A phase 2 study on CF patients also suggested that the efficiency of full-length protein expression restoration by ataluren would depend on the amount of transcript. mRNA concentration of at least 20% of wild type was needed to result in the normalization of total chloride transport in a patient with W1282X mutation, while concentrations lower than 10% of wild-type *CFTR* mRNA were high enough to give positive results in patients with G542X mutation [29].

Another important thing to consider is the identity of the amino acid introduced at the premature stop codon located at a critical enzymatic site, which can affect the functionality of the expressed protein. Recent characterization of translational readthrough products from HEK293T cells with TGA nonsense mutations showed that ataluren exposure resulted in the predominant insertion of arginine (≈69%), which was followed by tryptophan (≈28%) and cysteine (≈0.7%) at this particular stop codon [130].

### 2.12. Examples of Diseases at Treating Which Ataluren Was Not Effective

Ataluren was not an effective treatment for seizures in children suffering from nonsense variants of Dravet syndrome (DS) or CDKL5 Deficiency Syndrome (CDD) [131]. DS, also known as Severe Myoclonic Epilepsy of Infancy (SMEI), is an epilepsy syndrome of infantile-onset, belonging to the group of rare diseases. The symptoms include treatment-resistant seizures starting during the first year of life, developmental psychomotor delay, and the development of neurological deficits. Around 80% of DS patients carry sodium channel α1 subunit gene (*SCN1A*) mutations, some of which are nonsense mutations (Y325X, R1407X, R1645X) [132]. CDD is an X-linked neurodevelopmental disorder also characterized by early onset epilepsy and severe intellectual disability. Around 15% of *CDKL5* mutations are premature termination codons [133]. A single-center double-blind, placebo-controlled crossover trial (NCT02758626) enrolled seven patients with DS and eight with CDD bearing a nonsense mutation in one allele. Children aged 2–12 years received ataluren (10, 10, and 20 mg/kg) or placebo for 12 weeks, followed by a 4-week washout and then another 12-week treatment with the other treatment. No drug-related serious adverse effects were observed. One CDD patient had moderate gastrointestinal tract symptoms such as burping, flatulence, and vomiting during the blinded phase of the study, and another CDD patient had elevated liver function enzymes twice during the open-label period. Secondary outcome measures, that is changes in convulsive and/or drop seizure frequency as well as changes in minor seizure types, were not different between the ataluren-treated group and placebo group. Exploratory objectives that assessed changes in cognitive, motor, and behavioral functions, as well as the quality of life were not either different for the two study groups. All these findings suggest that ataluren may not be effective in treating these two primary brain disorders possibly due to limited crossing of the human blood–brain barrier, which was only assessed using rats and mice models. However, study limitations such as small sample size, short treatment period, poor patient adherence, and not sensitive enough outcome measures used should be noted [131]. 

Phenylketonuria is an autosomal recessive inborn error of phenylalanine metabolism. Most patients carry mutations in the phenylalanine hydroxylase (PAH) gene, among which 10% are nonsense mutations [134]. The readthrough efficiency of four common *PAH* nonsense mutations (R111X, R243X, R261X, and G272X), all involving UGA stop codon, was tested in vitro. PTC124 (0.003–28.4 μg/mL) did not induce the full-length protein expression in any cell line tested (COS-7 and HEK293) [135].

Long-QT syndrome type 1 (LQT1) is caused by nonsense mutations R518X-KCNQ1 (TGA-TGTA) and Q530X-KCNQ1 (TAG-TAAG). As a result, the slowly activating delayed rectifier potassium current channel *I*_Ks_ function is lost. *I*_Ks_ together with *I*_Kr_ are responsible for late phase 2 and phase 3 repolarization of the human ventricular action potential, which in turn leads to heart rhythm disorders and even sudden death. The treatment of HEK293 cells transfected with constructs carrying stop codon mutations with PTC124 (0.028, 2.84, and 28.4 μg/mL) did not induce any full-length protein expression [136]. 

The activity of ataluren was also tested using isolated rabbit ventricular cardiomyocytes to assess its potential use in patients with nonsense mutations in the *SCN5A* gene encoding the α-subunit of the voltage-gated sodium channel in the heart. This protein is critical for the propagation of the electric impulses, and the loss of its functions is associated with isolated conduction disease and Brugada syndrome. Both acute and long-term effects were assessed using the perforated patch-clamp methodology and either freshly isolated or 48 h cultured cardiomyocytes, respectively. No changes were observed for ataluren concentration tested (4.8 μg/mL) in resting membrane potential, maximum upstroke velocity, action amplitude, or action potential duration at 20, 50, and 90% of repolarization [137]. 

Treatment with ataluren was not effective as a nonsense suppression therapy for peroxisome biogenesis disorder (PBD) patients [12]. PBDs are a group of multisystemic autosomal recessive disorders caused by PEX mutations affecting normal peroxisome assembly and metabolic activities. Approximately 80% of PBD can be classified as the Zellweger spectrum (PBD-ZSD), while the remaining cases can be classified as rhizomelic chondrodysplasia punctata type 1 (RCDP1). The majority of PBD-ZSD patients (over 90%) have mutations in a group of five *PEX* genes, which are crucial for normal peroxisome assembly and function. Approximately 15% of these mutations are nonsense mutations [138]. Even more attractive for nonsense suppressor therapies is RCDP1, in case of which 60% of mutant alleles are *PEX7* L292X nonsense mutations [139,140]. However, ataluren showed no activity in PBD-patient skin fibroblasts producing stable PEX2 or PEX12 nonsense transcripts, nor in Chinese hamster ovary ZR-82 cells with a Pex2 nonsense allele (R123X). The drug was not able to promote translational readthrough of *PEX7* nonsense mutations (L292X) found in RCDP1 patients, either [12].

Ataluren was not either effective in reading-through premature stop codons in an obesity-associated gene encoding melanin 4 receptor (MC4R) in the hypothalamus. MC4R deficiency is the commonest known monogenic obesity disorder resulting in increased lean body mass, bone mineral density, and linear growth as well as hyperphagia and severe hyperinsulinemia [141]. Over 150 different *MC4R* mutations have been identified, some of them being nonsense mutations [142,143]. Treatment of COS-7 cells transfected with W16X MC4R mutation (TGA-A) with two ataluren concentrations (7.5 and 75 μg/mL) did not cause any increase in the total protein expression as measured by a total ELISA. What is more, the concentration of 75 μg/mL resulted in reduced expression of full-length protein in cells transfected with wild-type MC4R due to toxic effects at so high concentration [143].

## 3. Conclusions

Gene-independent therapies are attractive solutions to treat nonsense-mutation-based diseases since they can target any gene with premature termination codon characterized by favorable stop codon identity and its sequence context. Chemical stimulation of the PTC-readthrough by ataluren gave promising results in in vitro experiments and first clinical trials. PTC124 can be taken orally, and it was shown to have a good safety profile, better than traditional aminoglycosides, as evidenced by numerous studies. Even though the results of clinical trials in CF patients are inconclusive, recent evidence suggests that ataluren treatment can modestly slow disease progression in patients with nonsense mutation DMD, even though the primary endpoints of some clinical trials were not met [144]. The unsatisfactory performance can be partially explained by the samples’ heterogeneity in terms of different ages (which plays a critical role in disease progression and affects the patient’s response), mutation type (which affects DMD phenotype), and baseline characteristics. Another important issue is the limitations of the outcome measures. Immunohistochemistry and Western blot assays are known to have questionable reproducibility and reliability [145]. In most DMD clinical trials, the primary clinical endpoint used was the 6MWD, which was shown to be motivation-dependent [146]. Stronger evidence concerning ataluren’s efficacy is needed and hopefully, the results of the currently running long-term randomized, double-blind, placebo-controlled study (NCT03179631), which is to be completed in September 2023, will be unquestionably positive. Taking into account all the limitations of readthrough therapy, there is an increasing demand for alternative approaches. Those based on nucleic acids have many advantages. They are characterized by known mechanisms of action, sequence specificity [2], and a dose–response effect [144]. In 2016, the FDA approved the first drug for DMD, which is an antisense oligonucleotide eteplirsen with exon-skipping activity [147]. Upon binding to its target pre-mRNA, an antisense oligonucleotide is able to affect the splicing machinery to splice out PTC-bearing exon(s) from the mRNA transcript, which leads to the expression of a shorter, but functional protein. However, the exon-skipping transcript may also encode a non-functional protein, if regions involved in e.g., catalytic functions are lost, which is a significant disadvantage of this approach [2,148]. Moreover, since antisense nucleotides skip specific exons (e.g., eteplirsen and drisapersen omit exon 51, casimersen omits exon 45), such an approach requires more than one nucleic acid molecule to be developed in order to cover more than one group of patients depending on the PTC position. Consequently, this would result in long and expensive preclinical and clinical trials of each individual sequence-specific antisense nucleotide, since each of them will be chemistry-unique [148]. A nucleic acid-based technology characterized by a simple design that could also be used to correct nonsense mutations is a groundbreaking Cluster Regularly Interspaced Short Palindromic Repeats (CRISPR) gene editing. It is based on sequence-specific endonucleases cutting both strands of a DNA duplex in the vicinity of the site to be edited, which is followed by the endogenous DNA repair mechanism introducing changes in the nucleotide sequence [13]. Alternatively, deactivated versions of the nuclease conjugated with an adenosine deaminase can be used. These base editors can even target a mutated mRNA [2]. CRISPR gene editing has drawbacks, too. It is prone to off-target effects since mismatches in the complementary nucleotide sequence can be tolerated [13]. Despite their limitations, the three above-mentioned approaches are invaluable, since they target the underlying cause of the disease, not just its symptoms, and hopefully more and more safe and effective therapeutics able to suppress disease-causing PCTs will be approved for the treatment saving precious lives. 

## Figures and Tables

**Table 1:** Pseudoxanthoma elasticum nonsense mutations given in order of decreasing full-length protein expression level induced by incubation with 5 μg/mL ataluren.

Mutation	mRNA Nucleotide Sequence	Mutation Frequency (%)
R1275X	UGA-C	<1
R1164X	UGA-C	10
R1141X	UGA-A	54
Q378X	UAG-A	<1
Q1143X	UAG-G	<1
R518X	UGA-G	1.2
R1398X	UGA-G	1.2

**Table 2:** The summary of cell-based reporter assays used to test ataluren readthrough activity.

Cell line	Construct	Nonsense Mutation and Its Position	Read-Out	Ataluren Concentration (μg/mL)
ADXC8	Fluc2P (stable)	TGA-G at 223	luciferase	0.0000028–28.4
AD293	β-galactosidase (stable)	TGA-G at 320	β-gal	0.028–28.4
	Rluc (transient)	TGA-G at 21	luciferase	0.00028–28.4
AD293	FLAG-tagged collagen VII (transient)	TAG-T (Q251X)	ELISA	0.028–28.4
	FLAG-tagged collagen VII (transient)	TGA-G (R578X)	ELISA	0.028–28.4
AD293	Keratin 6a-YFP fusion (transient)	TGA/TAG/TAA at 533	Western blot	0.85

## Data Availability

Data sharing not applicable.

## Supplementary Materials

The following are available online at https://www.mdpi.com/article/10.3390/ph14080785/s1, Table S1: Summary of clinical trials.
