# Supplementary material for: Ataluren—Promising Therapeutic Premature Termination Codon Readthrough Frontrunner
Source: Pharmaceuticals (Basel). 2021 Aug 9;14(8):785. doi: 10.3390/ph14080785 (PMC8398184; doi:10.3390/ph14080785)
Supplement: Supplementary file 1 [file pharmaceuticals-14-00785-s001.zip › pharmaceuticals-1314944-supplementary.pdf]

Table S1. Summary of clinical trials.

Trial Number	Phase	Time Period	Details
		POLYTHERAPY	
NCT02409004	1 completed	February 2015–March 2015	15 adults 1375 mg once daily
		DUCHENNE MUSCULAR DYSTROPHY	
NCT02369731	recruiting	April 2015–May 2025	360 subjects ≥ 2 years
NCT01182324	Retrospective completed	July 2010–June 2013	21 adults
NCT00264888 [39]	2a completed	December 2005–May 2007	38 boys ≥ 5 years; 28 days<br>TID [1]: 4, 4, 8 mg/kg; 10, 10, 20 mg/kg; 20, 20, 40 mg/kg
NCT00592553 [41]	2b completed	February 2008–December 2009	174 males ≥ 5 years; 48 weeks<br>TID: 10, 10, 20 mg/kg; 20, 20, 40 mg/kg
NCT00847379	2b terminated	January 2009–May 2010	173 males ≥ 5 years; 96 weeks<br>TID: 20, 20, 40 mg/kg
NCT00759876	2 terminated	August 2008–May 2010	36 males; 89 weeks<br>TID: 10, 10, 20 mg/kg
NCT01009294	2 terminated	January 2010–March 2010	6 subjects ≥ 7 years; 48 weeks<br>TID: 20, 20, 40 mg/kg
NCT03648827	2 completed	December 2018–October 2020	20 males 2–7 years; 40 weeks<br>TID: 10, 10, 20 mg/kg
NCT02819557	2 completed	June 2016–February 2018	14 subjects ≥ 2 to < 5 years; 52 weeks<br>TID: 10, 10, 20 mg/kg
NCT03796637	2 completed	April 2019–May 2019	6 males<br>TID: 10, 20, 20 mg/kg
NCT04336826	2 not yet recruiting	June 2021–June 2023	6 children 6 months–2 years; 52 weeks<br>TID: 10, 10, 20 mg/kg
NCT01247207	3 enrolling by invitation	November 2010–December 2021	160 males<br>TID: 10, 10, 20 mg/kg
NCT01557400	3 completed	May 2012–January 2018	94 males; 240 weeks<br>TID: 10, 10, 20 mg/kg
NCT018264487 [54]	3 completed	March 2013–August 2014	230 boys 7–15 years; 48 weeks<br>TID: 10, 10, 20 mg/kg
NCT02090959	3 terminated	March 2014–June 2018	219 boys 7–14 years; 144 weeks<br>TID: 10, 10, 20 mg/kg
NCT031179631	3 active not recruiting	July 2017–October 2023	250 ≥ 5 years; 72 weeks<br>TID: 10, 10, 20 mg/g
		CYSTIC FIBROSIS	
NCT00234663	2 completed	September 2005–August 2006	24 subjects ≥ 18 years<br>TID: 4, 4, 8 mg/kg (14 days),<br>then 10, 10, 20 mg/kg (14 days)
NCT00237380 [29]	2 completed	November 2005–May 2006	24 adults 18–56 years; 28 days<br>TID: 4, 4, 8 mg/kg; 10, 10, 20 mg/kg
NCT00351078 [37]	2 completed	December 2006–July 2007	19 adults 19–57 years; 12 weeks<br>TID: 4, 4, 8 mg/kg (14 days),<br>then 10, 10, 20 mg/kg (14 days)
NCT00458341 [38]	2 completed	March 2007–February 2008	30 children; 6–18 years<br>TID: 4, 4, 8 mg/kg (14 days),

			then 10, 10, 20 mg/kg (14 days)
NCT00803205 [33]	3 completed	September 2009–November 2011	238 subjects ≥ 6 years; 48 weeks TID: 10, 10, 20 mg/kg
NCT01140451	3 completed	August 2010–December 2013	191 subjects ≥ 6 years; 96 weeks TID: 10, 10, 20 mg/kg
NCT02107859	3 terminated	March 2014–June 2017	61 subjects ≤ 6 years; 192 weeks TID: 10, 10, 20 mg/kg
NCT02139306 [75]	3 completed	August 2014–November 2016	279 subjects > 6 years; 48 weeks TID: 10, 10, 20 mg/kg
NCT02456103	3 terminated	August 2015–June 2017	246 subjects ≤ 6 years; 96 weeks TID: 10, 10, 20 mg/kg
NCT03256968	4 completed	January 2017–December 2018	1 subject ≥ 6 years
NCT03256799	4 completed	March 2017–February 2018	1 subject ≥ 19 years; 48 weeks
EPILEPSY			
NCT02758626 [131]	2 active, not recruiting	November 2016–March 2021	16 boys 2–12 years; 12 weeks
HEMOPHILIA A AND B			
NCT00947193	2 terminated	October 2009–August 2011	13 adults; 14 weeks TID: 5, 5, 10 mg/kg or 10, 10, 20 mg/kg
METHYLMALONIC ACIDEMIA			
NCT01141075	2 terminated	July 2010–November 2011	11 subjects ≥ 2 years TID: 5, 5, 10 mg/kg (cycle 1) 10, 10, 20 mg/kg (cycle 2)
COLORECTAL CANCER, ENDOMETRIUM CANCER			
NCT04014530	1 (colorectal cancer) 2 (endometrium cancer) recruiting	August 2019–August 2023	47 adults
ANIRIDA			
NCT02647359	2 completed	January 2016–January 2021	39 subjects ≥ 2 years; 144 weeks TID: 10, 10, 20 mg/kg
NCT04117880	2 withdrawn	December 2018–January 2021	0 subjects ≥ 2 years TID: 20, 20, 40 mg/kg

[1] TID three time per day.
